# Photoacoustic phase and intensity dual-modality imaging for characterization of plaque viscoelasticity and lipid composition: a feasibility study

**DOI:** 10.1117/1.JBO.32.S1.S14904

**Published:** 2026-10-28

**Authors:** Yuxuan Liang, Yiting Tan, Ao Jiang, Heng Zhang, Pingping Wang

**Affiliations:** a Shaoxing University, School of Medicine, Department of Clinical Medicine, Shaoxing, China; b South China Normal University, Institute of Laser Life Science, MOE Key Laboratory of Laser Life Science, Guangzhou, China

**Keywords:** atherosclerotic plaque, photoacoustic phase imaging, photoacoustic intensity imaging, viscosity-elasticity ratio, lipid component

## Abstract

**Significance:**

Rupture of atherosclerotic plaque is the most important cause of sudden death in acute myocardial infarction. Plaque stability is closely related to mechanical characters and histological composition. A dual-modality technique, combining photoacoustic phase imaging (PPI) and intensity imaging (PAI), was developed providing viscoelasticity and lipid component of plaques simultaneously.

**Aim:**

We aimed to investigate the feasibility of PPI to identify thin-cap fibrous plaques by detecting viscosity-elasticity ratio (VER), to further examine the correlation between VER and the major components of plaque, and ultimately to verify the accuracy of PPI-PAI in assessing plaque types.

**Approach:**

PPI-PAI experiments were performed on the aortas of 18 Apolipoprotein E-knockout (ApoE) mice fed with high-fat/high-cholesterol diet. After each experiment, the plaques at the target sites were stained with Oil Red O, Masson, and F4/80 staining to verify the accuracy of the imaging and to further investigate the correlations between VER and lipid core area, fibrous cap thickness, and macrophage content. Finally, conventional statistical methods, such as linear analysis, were used to summarize the experimental data and draw general conclusions.

**Results:**

PPI enables quantitative VER detection of plaques, with thin-cap fibrous plaques exhibiting the highest VER ($3.77\times 10^{-5}∼4.68\times 10^{-5}\;s$). There is a high positive correlation between VER and lipid cross-section area ($R^{2}=0.81$, p<0.0001) and macrophage content ($R^{2}=0.75$, p<0.0001) in lipid plaque lesions. Likewise, in fibrous plaques, a strong negative correlation ($R^{2}=0.71$, p<0.0001) is demonstrated between VER and fibrous cap thickness; and a high positive correlation ($R^{2}=0.12$, p=0.02) between VER and macrophage content. As lipid plaques and thick-cap fibrous plaques may have the same VER values, the combination of PPI and PAI can effectively identify plaque types.

**Conclusions:**

PPI facilitates the characterization of thin-cap fibrous plaques by VER measurements, and further combination with PAI can improve the detection rate of distinct plaque types.

## 1 Introduction

The composition of atherosclerotic plaques is an important determinant in the progression of thrombus-mediated acute coronary syndromes.^1^^,^^2^ The typical constituent structures of vulnerable plaques include a thin fibrous cap (minimum cap thickness <65  μm), a large necrotic core, and local inflammatory reaction.^3^^,^^4^ In addition to these morphological and compositional features, the mechanical properties of atherosclerotic plaques play a vital role in plaque rupture.^5^^,^^6^ In the early stage (lipid plaques) of atherosclerosis, the degree of lipid accumulation affects the mechanical properties of plaques.^7^^,^^8^ When developing into middle stage (thick-cap fibrous plaques), the mechanical strength of plaques is primarily determined by the fibrillar collagen content of the extracellular matrix.^9^ However, in the late stage (thin-cap fibrous plaques), collagen degradation is directly associated with an increased risk of plaque rupture.^10^^,^^11^ In a diseased artery, the rupture of most plaques often occurs in regions with low elasticity and high viscosity, which are unable to bear the high circumferential stress due to the pulsating blood pressure.^12^^,^^13^

Various biomechanical studies are currently employed to detect high-risk plaque in coronary syndromes, mainly including ultrasound elastography, optical coherence elastography, and laser speckle imaging.^14^^–^^16^ Elasticity and viscosity are two crucial physical parameters for mechanical characterization of atherosclerotic plaques.^17^ As atherosclerosis develops, the viscosity and elasticity of plaques tend to change conversely, as a result of the proportion changes in plaque composition [Fig. 1(a)].^18^^,^^19^ In addition, a single mechanical parameter (elasticity or viscosity) is not able to accurately describe the mechanical properties of the plaques.^20^ Viscosity-elasticity ratio (VER) characterization has the effect of magnification of the relative changes in elasticity and viscosity of atherosclerotic plaques. Thus, measuring the VER will realize the high-sensitivity detection of composition change of plaques.

**Fig. 1:**
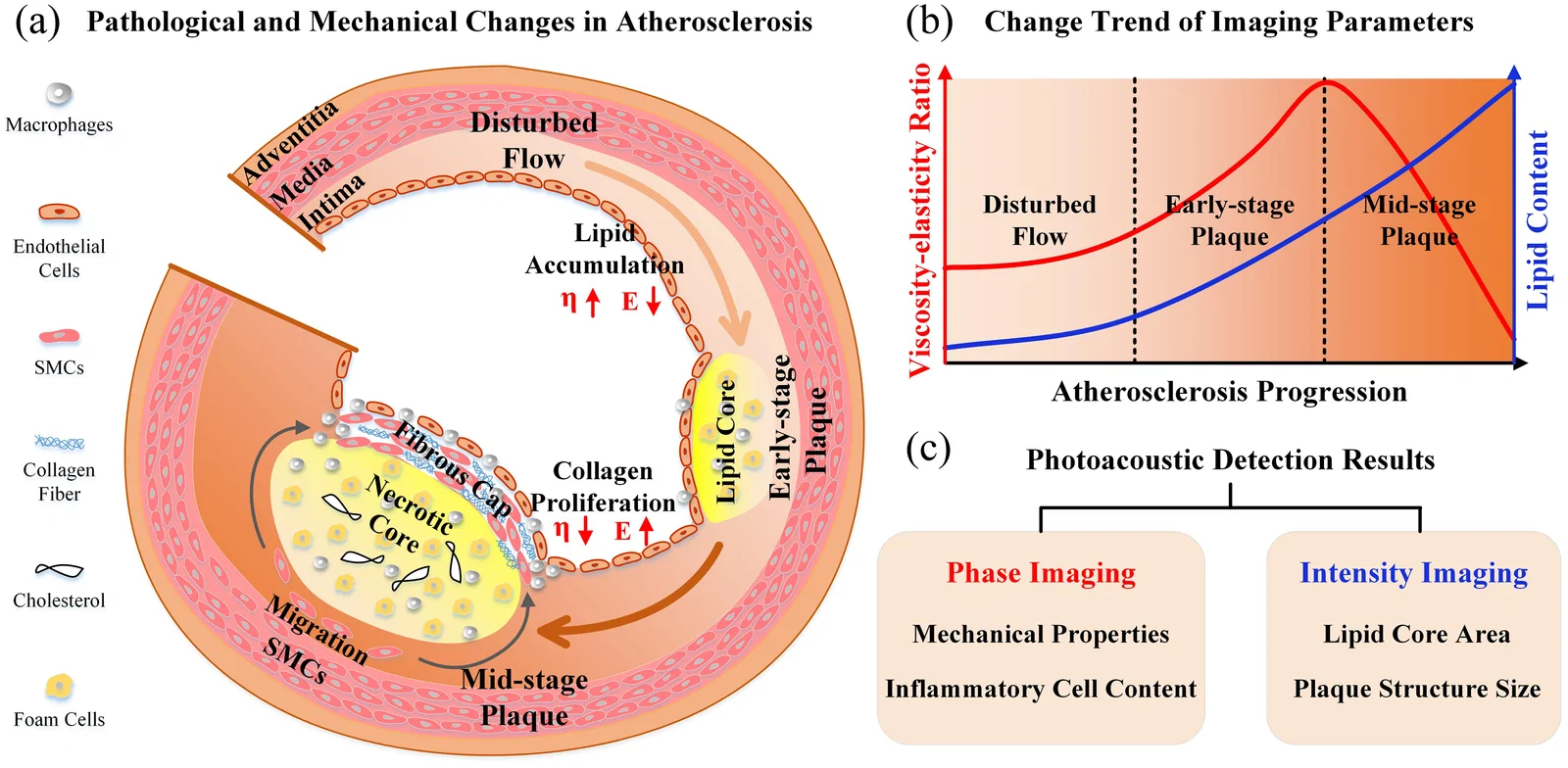
Pathological and mechanical changes in early- to mid-stage atherosclerosis, and the detection of VER and lipid content of plaque by PA technique. (a) At areas of disturbed blood flow, endothelial cells are activated, and adherens junctions dissociate resulting in lipid accumulation in the vessel wall (η increases; E decreases). Recruitment of macrophages, foam cell formation, and adventitial smooth muscle cell (SMC) proliferation contribute to plaque progression (η decreases; E increases). (b) As atherosclerotic plaques develop, the VER increases first and then decreases, whereas the lipid content keeps increasing. (c) Basic information about plaques obtained through PA technique. η, viscosity coefficient; E, Young’s modulus.

Here, we investigate a quantitative biomechanical method, photoacoustic phase imaging (PPI), for obtaining information about VER of atherosclerotic plaques. Irradiated with an excited pulse laser, biological tissue produces photoacoustic (PA) wave, and a phase lag behind it existed owing to the damping effect caused by biological viscoelasticity.^21^ The phase delay is directly related to the viscoelastic properties of tissue.^21^ The VER image can be reconstructed from the recorded phase delay between the PA signals and the excitation pulses.^21^^,^^22^ We have demonstrated the capability of PPI in viscoelastic feature detection, which has been applied in tissue differentiation, tumor detection, and early atherosclerosis characterization.^23^^–^^25^ However, during the early to middle stages of plaque development, the same VER values may be observed in both the lipid and fibrous plaques as the formation of the fibrous cap leads to a decrease in VER values. Nevertheless, as the plaque progresses pathologically, the lipid content continues to increase [Fig. 1(b)]. Photoacoustic intensity imaging (i.e., absorption imaging, PAI), which utilizes the intensity information of PA signals, allowed localization and quantification of lipid content in atherosclerosis.^26^^,^^27^ In this article, we combine PPI and PAI for providing information on the VER and lipid content of plaques, and finally achieved the detection of atherosclerotic plaques [Fig. 1(c)].

The aim of this study is first to establish a complete calibration framework incorporating system adjustment, phantom validation, and rheometer reference measurements to achieve quantitative determination of the VER. Second, mice aortic plaque samples are used to investigate whether changes in the composition of atherosclerotic plaques can cause the changes in VER index and further validate the relationship between VER and lipid core area, and cap thickness and macrophage content. Finally, the feasibility and accuracy of PPI-PAI system for identifying plaque types are evaluated.

## 2 Materials and Methods

### 2.1 Experimental Setup

The schematic of PPI-PAI microscopy system is shown in Fig. 2(a). For the PPI subsystem, a quasi-continuous laser (DS20HE-1064D/R, PHOTONICS) with pulse width of 22 ns and wavelength of 1064 nm operating at the repetition frequency of 50 KHz was used as the excitation source. In addition, for the PAI subsystem, an optical parametric oscillator laser (NT200 Series Laser, Ekspla, Vilnius, Lithuania) delivers 8 ns laser pulses at a wavelength of 1700 nm was used as the radiation source with a repetition rate of 2.5 kHz. The two laser beams were aligned using a dichroic mirror and then collimated and focused onto the sample surface via a 4× microscope objective (numerical aperture, NA=0.1), thereby generating PA signals in the tissue. The laser energy density incident on the tissue surface was controlled below $15\;\mathrm{mJ}/{\mathrm{cm}}^{2}$ during the experiments, which was lower than the American National Standards Institute (ANSI) laser safety limits ($20\;\mathrm{mJ}/{\mathrm{cm}}^{2}$).^28^ During the experiment, the PA signals were received in reverse mode and acquired via two channels: The PPI signals were carried out by a custom ultrasound transducer 1 (${\mathrm{UT}}_{1}$ with a center frequency of 50 kHz) to receive signals. The sensing area was positioned near the laser focal point on the tissue, at an angle of ∼45  deg to the plane and close to the sample. The signals were then transmitted to a preamplifier (SR552, Stanford Research Systems, Sunnyvale, California, United States) and fed into a lock-in amplifier (OE2031, SYSU Scientific Instruments, Guangzhou, China) to obtain the phase delay of the PPI signals. In addition, the PAI signals were received by a custom hollow focused ultrasound transducer 2 (${\mathrm{UT}}_{2}$, 30 MHz center frequency, 90% ∼6  dB bandwidth). The acquired signals were transmitted to a wide-bandwidth low-noise amplifier (Ha2, Precision Acoustics LTD, Dorset, United Kingdom), digitized and collected by a high-speed data-acquisition card (M-3i. 4120, Spectrum, Grosshansdorf, Germany) at a sampling rate of 250 MHz, and finally stored in the computer. Finally, the phase delay and absorption intensity of the PPI and PAI signals were analyzed using a computer. The system was operated via a custom-made LABVIEW program (National Instruments, Austin, Texas, United States). MATLAB software (Mathuorks, Natick, Massachusetts, United States) was used for image construction and index measurement. Following calibration of system and simulation samples, the calibration equation relating PPI-derived VER and rheometer-measured VER was ${\mathrm{VER}}_{R}=2.69{\mathrm{VER}}_{\mathrm{PA}}+0.17\times 10^{-6}$ ($R^{2}=0.90$, p<0.0001). This equation enabled application to subsequent tissue measurements. The detailed phase calibration workflows and rheometer (Anton Paar MCR 302e) parameter settings are described in Part S1 of the Supplementary Material and Part S2 of the Supplementary Material, respectively. In addition, the details regarding the PAI signal processing and lipid characterization are shown in Part S3 of the Supplementary Material.

### 2.2 PPI Principle

The basic principle of PPI is shown in Fig. 2(b). Due to the damping effects of the tissue, the generated PA signals generated to have a phase delay relative to the laser trigger, and the magnitude of this phase delay was directly related to the viscoelastic properties of the tissue. In the rheological Kelvin–Voigt model, the relationship between the phase delay δ and the viscosity-to-elasticity ratio (η/E) is δ=arctan ηω/E, where η is the viscosity coefficient, ω is the modulation frequency, and E is the Young’s modulus.^21^^,^^22^ The detailed derivation is shown in Part S4 of the Supplementary Material. Notably, although the numerical magnitude of VER is utilized for comparative diagnostic evaluation, the parameter physically denotes a relaxation time and bears units of seconds (s). As the focal depth of the laser in the tissue is approximately 100  μm, precisely, the PA information reflects the average VER value in the depth range of 0 to 100  μm.

**Fig. 2:**
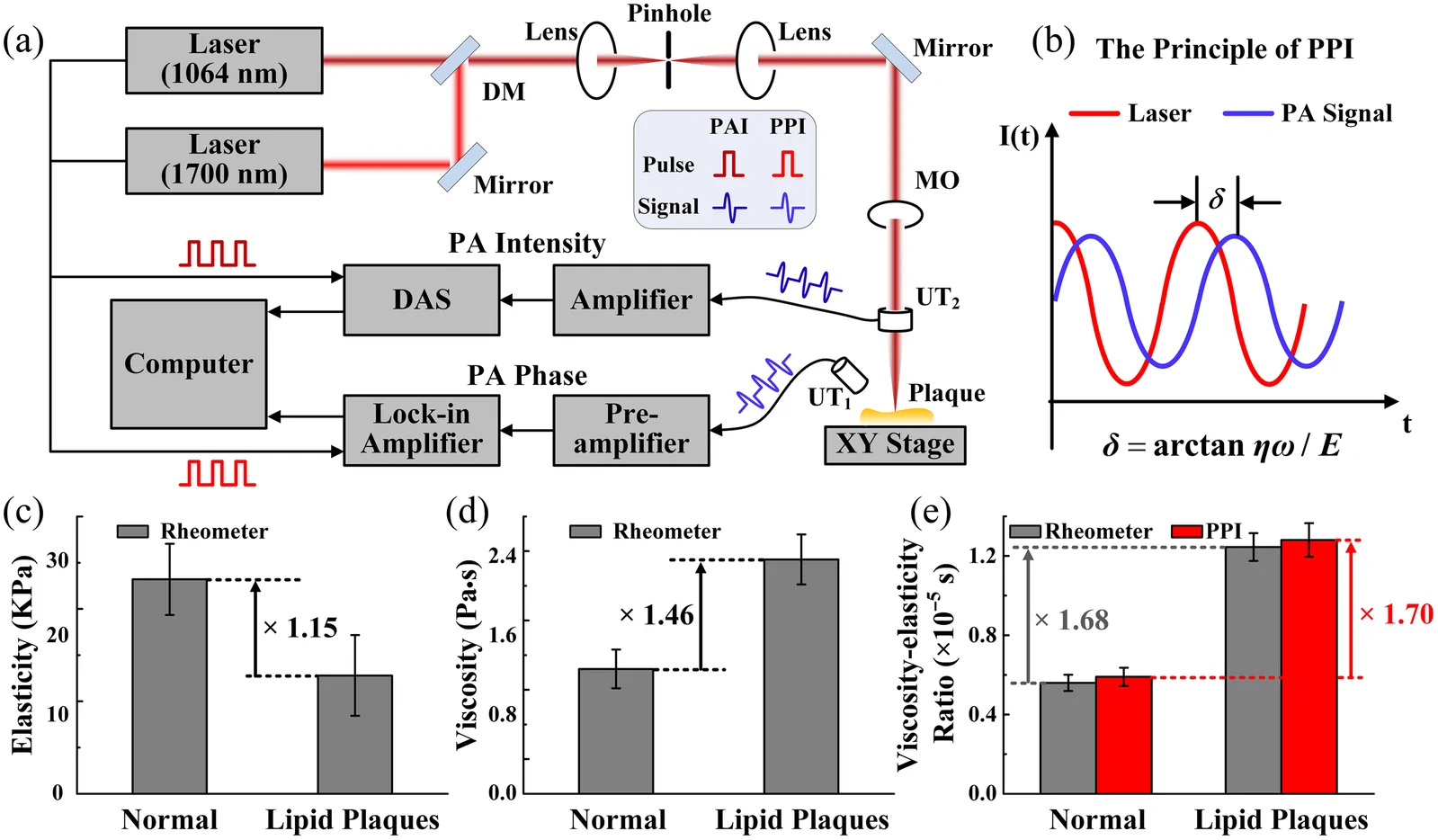
Imaging system and detection mechanism. (a) Schematic of the PPI-PAI system. (b) Principle of the PPI for detecting the VER value of plaques though the phase delay of PA signal. η, viscous coefficient; E, Young’s modulus; δ, PA phase delay; ω, laser modulation frequency; I(t), intensity; t, time; DAS, data-acquisition system; DM, dichroic mirror; MO, microscope objective; ${\mathrm{UT}}_{1,2}$, ultrasonic transducer.

### 2.3 Experimental Protocol

This study was approved by the Animal Study Committee of South China Normal University College of Biophotonics in Guangdong, China. Eighteen Apolipoprotein E-knockout (ApoE) mice (C57BL/6 background) were used in the cross-sectional plaque experiment, and received 0, 4, 8, 12, 20, or 24 weeks of high-fat/high-cholesterol (HFC) diet, respectively (3 mice per stage). At the end of each feeding stage, the mice were euthanized with an overdose of pentobarbital (3%, 120  mg/kg). The thoracic aorta of each mouse was excised and flushed with PBS (PH=7.4) and was promptly examined with the PPI-PAI system.

After the experiments and subsequent formalin fixation, the marked arterial segments were dissected. For all vessels, sections of atherosclerotic plaques were sliced near the center of the marked segment for lipid with Oil Red O, fibrous cap with Masson, and macrophages with F4/80 Immumohistochemical staining. Histology experiments were performed by investigators blinded to the imaging results. Histopathological assessment was performed independently by two observers. Plaques were ultimately classified into three types: lipid plaques (lipid deposition without a fibrous cap), thick-cap fibrous plaques (fibrous cap thickness≥65  μm), and thin-cap fibrous plaques (fibrous cap thickness<65  μm). Details of the assessment workflow and classification outcomes are provided in Part S5 of the Supplementary Material.

### 2.4 Statistical Analysis

Continuous data are presented as mean ± standard deviation (SD) or median (interquartile range), as appropriate. Bar, line, and box-and-whisker plots were generated in Origin 2020 (OriginLab Corporation, Northampton, Massachusetts, United States) solely for descriptive visualization of temporal trends; no between-group comparisons were performed on these plots. The association between VER and histological findings (lipid area, fibrous cap thickness, and macrophage content) was assessed by Pearson’s correlation and simple linear regression in Origin 2020. Correlation coefficients (r) are reported with 95% confidence intervals (CIs), and the regression equation is reported with slope, intercept, and coefficient of determination ($R^{2}$). Agreement between PPI and rheometer was assessed by Bland–Altman analysis in GraphPad Prism version 8.0 for Windows (GraphPad Software, San Diego, California, United States). The mean difference (bias) quantifies systematic deviation and is reported with its 95% CI. The 95% limits of agreement, calculated as bias ±1.96× SD of the differences, define the range within which 95% of differences are expected to lie. Proportional bias was examined by regressing the differences on the means. Diagnostic performance was assessed via receiver operating characteristic (ROC) analysis following leave-one-out cross-validation. Analyses were performed in GraphPad Prism. The area under the ROC curve (AUC) is reported with its 95% CI, and the optimal cut-off value is determined by maximizing the Youden’s index, with the corresponding sensitivity and specificity reported. A two-sided p value<0.05 was considered statistically significant. Notably, all plaque-associated statistical analyses adopted plaque-bearing cross-sections as observational units instead of individual animals (sample sources detailed in Part S6 of the Supplementary Material). All results were interpreted at the plaque lesion level without extrapolation to the animal level.

### 2.5 Principle of Plaque VER Detection

To demonstrate the magnification mechanism of PPI in detecting the mechanical properties of atherosclerotic plaques, the elasticity, viscosity and VER of plaques were measured. Figures 2(c)–2(e) show the rheometer and PPI results of the normal arteries (0 week of HFC diet) and the lipid plaques (12 weeks of HFC diet). According to rheometer measurements, the elasticity (Young’s modulus) of normal blood vessels is 1.15 times that of plaques, whereas the viscosity (viscosity coefficient) of plaques is 1.46 times that of normal blood vessels. Furthermore, the VER values of the plaques measured by rheometer and PPI were 1.68 times and 1.70 times those of normal blood vessels, respectively. The experimental results indicated that the VER measured by PPI magnified the opposing changes in viscosity and elasticity that occurred during atherosclerosis, thus enabling effective and accurate detection of plaque pathology.

## 3 Results

### 3.1 Simulated Sample Experiment

The phantom experiment was conducted to evaluate the spatial resolution and imaging depth of the PPI-PAI system. A blade was embedded in a gelatin (48,722, Sigma-Aldrich, Burlington, Massachusetts, United States) phantom and imaged using this system. The distributions of PPI and PAI intensity are shown in Figs. 3(a) and 3(b), respectively. The lateral resolutions of the PPI and PAI were 11.4 and 11.8  μm, respectively, which were defined as the full-width-at-half-maximum (FWHM) of the line spread function (LSF). Figure 3(c) shows that the axial resolution of the PAI, based on the FWHM of the profile, was estimated to be 34.9  μm. To demonstrate the imaging depth of the system, an iron wire was inserted into the fat for simulating the lipid. The iron wire diameter was about 0.3 mm. The measuring method was shown in the inserted schematic. The signal intensity of PAI decreased with increasing imaging depth, and the intensity of background noise remained almost unchanged, as demonstrated in Fig. 3(d). Obviously, the imaging depth of PAI could exceed 3 mm in fat. All the results suggested that the PPI-PAI system had the ability to detect the plaques.

**Fig. 3:**
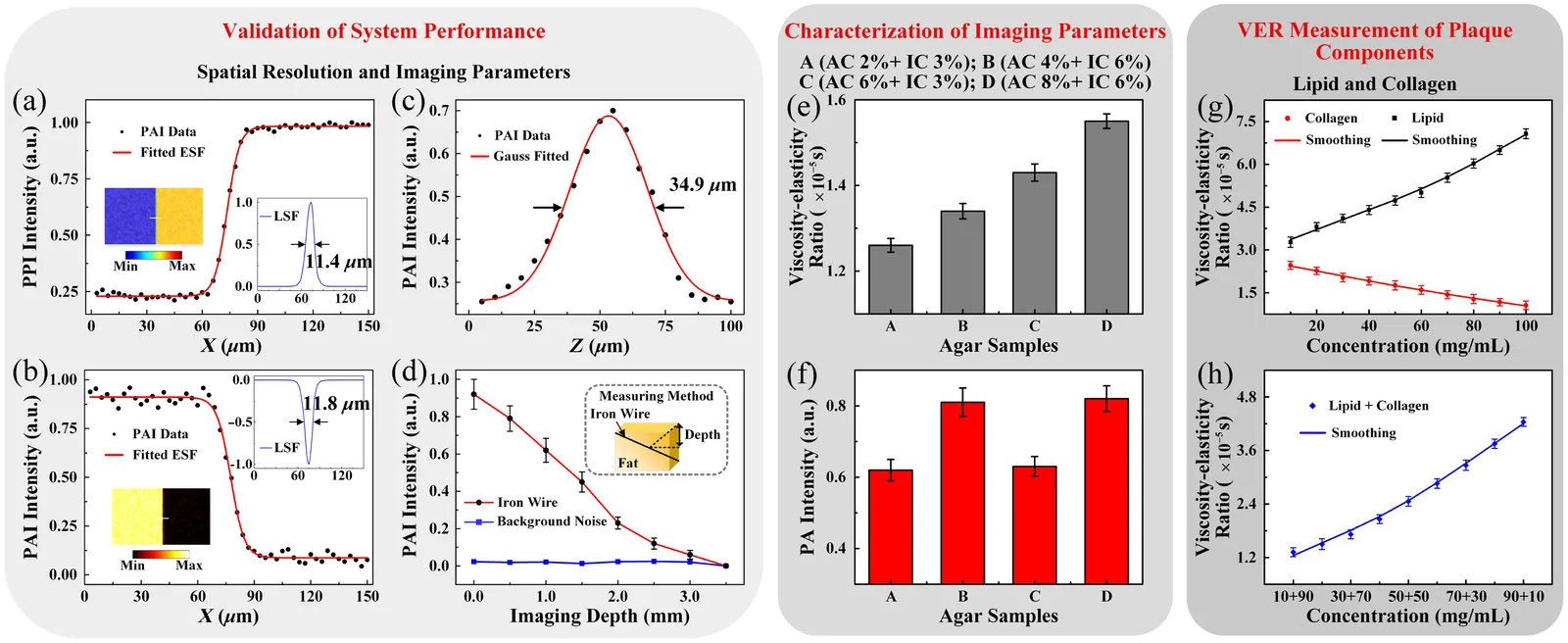
Simulated sample experiment. Panels (a) and (b) represent the lateral resolution of PPI and PAI, respectively. Panel (c) represents the axial resolution of PAI. (d) Imaging depth of the system. The inserted schematic shows the measurement method. ESF, edge spread function; LSF, line spread function. Panels (e) and (f) show the comparison of VER and PA signal intensity, respectively. The VER of (f) phantoms containing various concentrations of lipid or collagen and (g) phantoms mixed with different proportions of lipid and collagen.

The dual-parameter PPI-PAI images of tissue-mimicking phantoms with different VER and absorption are illustrated in Fig. 3. Four samples marked with “A,” “B,” “C,” and “D” contained agar with the concentrations of 2%, 4%, 6%, and 8%, respectively. The samples “A” and “C” were interfused of ink with proportion of 3%, and the samples “B” and “D” were interfused of ink with proportion of 6%. Figures 3(e) and 3(f) show the mean value and standard deviation of the VER and PA intensity amplitude of each sample. As the gelatin concentration increased, the VER value increased significantly [Fig. 3(e)]. However, the PA intensity increased with the increasing ink concentration, regardless of gelatin concentration [Fig. 3(f)]. The results indicated that the PPI and PAI imaging modalities were independent of one another, and that the VER and optical absorption contrast mechanism could compensate for each other’s deficiencies.

As the main components in atherosclerotic lesions, lipid and collagen were directly related to the mechanical properties of plaques and played important roles in the pathological progression of plaques. The ability of PPI to differentiate plaque components in tissue-mimicking phantoms mixed with gelatin was analyzed. Figure 3(g) shows the relationship between the content of lipid or collagen and the VER, as well as the VER of phantoms containing different proportions of lipid and collagen [Fig. 3(h)]. VER increased as lipid content increased; however, it decreased as collagen content increased. In the case of mixed lipid-collagen samples, the VER value increased as the lipid content increased and the collagen content decreased. The results indicated that the VER detection of plaque composition was accurate and appropriate.

### 3.2 VER Measurement in Different Types of Plaques

To investigate whether PPI had the ability to distinguish between different types of plaques, the VER results of plaques in mice fed with HFC diet for different periods are shown in Fig. 4. The VER of normal vessel ($\mathrm{VER}∼2.05\times 10^{-5}\;s$; mouse fed with HFC diet for 0 weeks) [Fig. 4(a)] differed significantly compared to lipid plaque ($\mathrm{VER}∼3.21\times 10^{-5}\;s$; mouse fed with HFC diet for 12 weeks) [Fig. 4(b)]. Oil Red O stain results showed the lipid accumulation in the fatty arteries. Compared with the thick-cap fibrous plaque ($\mathrm{VER}∼1.56\times 10^{-5}\;s$; mouse fed with HFC diet for 20 weeks) [Fig. 4(c)], higher average VER values were found in the thin-cap fibrous plaque ($\mathrm{VER}∼4.22\times 10^{-5}\;s$; mouse fed with HFC diet for 24 weeks) [Fig. 4(d)]. Furthermore, Masson staining results revealed the decrease of collagen in thin-cap in contrast to the thick-cap fibrous plaque. In addition, among all the plaque types, thin-cap fibrous plaque exhibited the highest VER, ∼2.06 times that of normal vessel, suggesting that this stage represented a late stage of plaque formation. Overall, these results demonstrated the potential of PPI for distinguishing plaque types.

**Fig. 4:**
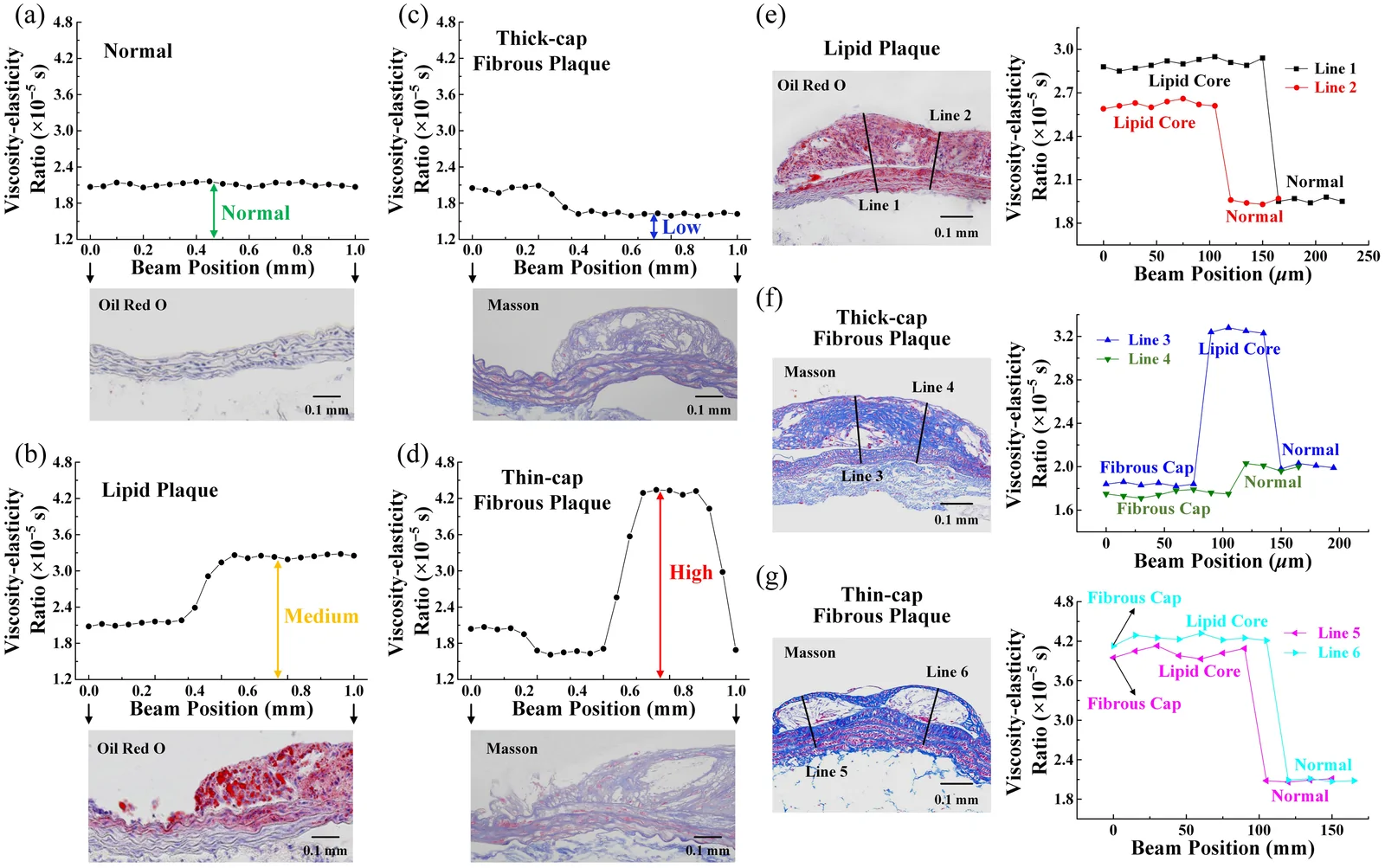
VER detection of different types of plaques. Variation in VER as illumination beam scans laterally across (a) normal vessel, (b) lipid plaque, (c) thick-cap fibrous plaque, and (d) thin-cap fibrous plaque. Variation in VER as illumination beam scans axially across (e) lipid plaque, (f) thick-cap fibrous plaque, and (g) thin-cap fibrous plaque.

Currently, PPI only yielded two-dimensional images limited to superficial tissues (0 to 100  μm), without capturing the axial variation of plaque viscoelastic properties. Axial scanning of plaques was therefore conducted to verify the discriminatory performance of PPI for distinct plaque components, as presented in Fig. 4. For lipid plaques [Fig. 4(e)], the thicknesses of the lipid cores at lines 1 and 2 were measured to be ∼160 and 112  μm, respectively, with corresponding average VER values of $2.90\times 10^{-5}$ and $2.62\times 10^{-5}\;s$. This variation was likely attributable to the compositional heterogeneity of the lipid cores across plaques of differing sizes. In addition, as lipid content increased, VER tended to increase. For thick-cap fibrous plaques [Fig. 4(f)], the fibrous cap thickness at lines 3 and 4 was ∼96 and 124  μm, respectively, with corresponding average VER values of $1.84\times 10^{-5}$ and $1.75\times 10^{-5}\;s$. Reduced fibrous cap thickness corresponded to increased VER. Beneath the fibrous cap at line 3, approximately four sampling points exhibited relatively elevated VER, suggesting the presence of a lipid core. For thin-cap fibrous plaques [Fig. 4(g)], the fibrous cap thickness at lines 5 and 6 reached ∼14 and 9  μm, respectively. As the scanning step size was set to 15  μm, only a single scanning point was obtained within the fibrous cap for each lesion, with VER values of $4.35\times 10^{-5}$ and $4.48\times 10^{-5}\;s$ at lines 5 and 6, respectively. The thicknesses of the lipid core beneath the fibrous caps at lines 5 and 6 were 83 and 115  μm, respectively, with corresponding average VER values of $4.46\times 10^{-5}$ and $4.65\times 10^{-5}\;s$. Notably, the VER values at the plaque surface of lines 5 and 6 were $4.41\times 10^{-5}$ and $4.58\times 10^{-5}\;s$, respectively, and both exceeded the values acquired in the fibrous cap regions. This suggested that VER detection via PPI offered an integrated assessment of the superficial tissue ranging from 1 to 100  μm thick. By contrast, normal vascular tissue exhibited relatively homogeneous mechanical properties for lipid plaques, thick-cap fibrous plaques, and thin-cap fibrous plaques. Nevertheless, the VER value of the normal tissue associated with thin-cap lesions ($\mathrm{VER}∼2.09\times 10^{-5}\;s$) was greater than that associated with thick-cap lesions ($\mathrm{VER}∼2.00\times 10^{-5}\;s$). The normal tissue adjacent to lipid plaques exhibited the lowest VER values, at $∼1.95\times 10^{-5}\;s$. Although the differences were minor, this finding indicated that compositional changes occurred in the normal vascular wall during the pathological progression of plaques. Collectively, these findings demonstrated that the observed changes in VER were closely associated with variations in plaque composition.

### 3.3 Characterization of Lipid Plaques

The PPI-PAI system was able to characterize the lipid accumulation during lipid plaque lesions. Figure 5(a) shows the cross-section PPI and PAI images found in mice aortas of 0 to 12 weeks old, as well as the histological results. Lipids presented a strong absorption in PAI, and the size of lipid core measured by PAI corresponded well to the Oil Red O staining (the area enclosed by the dotted line in the images). Masson staining revealed that there was no fibrous cap in lipid plaques, and the F4/80 staining results were used to demonstrate the presence of macrophages. As the HFC diet period progressed, the lipid core area measured by PAI gradually increased, and the VER value measured by PPI also steadily rose. Plaques in the 4-week-old mice were early lesion with an average VER of $2.62\times 10^{-5}\;s$. The vessel walls of mice after 8 weeks had more mixed lipid, as indicated by histology, and showed an average VER of $2.84\times 10^{-5}\;s$. An advanced lipid region, where the VER ranged from $3.07\times 10^{-5}$ to $3.54\times 10^{-5}\;s$, corresponding to the increase of lipid content, was observed after 12 weeks. Figure 5(b) shows the lipid area, measured by PAI and histology, plotted against the duration of the HFC diet. In Fig. 5(c), linear regression analysis revealed a significant linear association between lipid cross-section area and VER at the plaque lesion level, with a slope of $39.23\times 10^{5}\%/s$ ($95\%\;\mathrm{CI}:34.53\times 10^{5}\%/s$ to $44.71\times 10^{5}\%/s$), intercept of −84.19% (95% CI: −91.45% to −76.49%), and coefficient of determination $(R^{2})=0.81$, p<0.0001, based on 30 plaque sections (n=30) obtained from 12 mice fed a HFC diet for 0 to 12 weeks. These results indicated that, during the lipid plaque stage, lipid deposition could be directly characterized by PAI; it could also be quantitatively assessed through PPI measurement, and the VER values were closely correlated with lipid content.

**Fig. 5:**
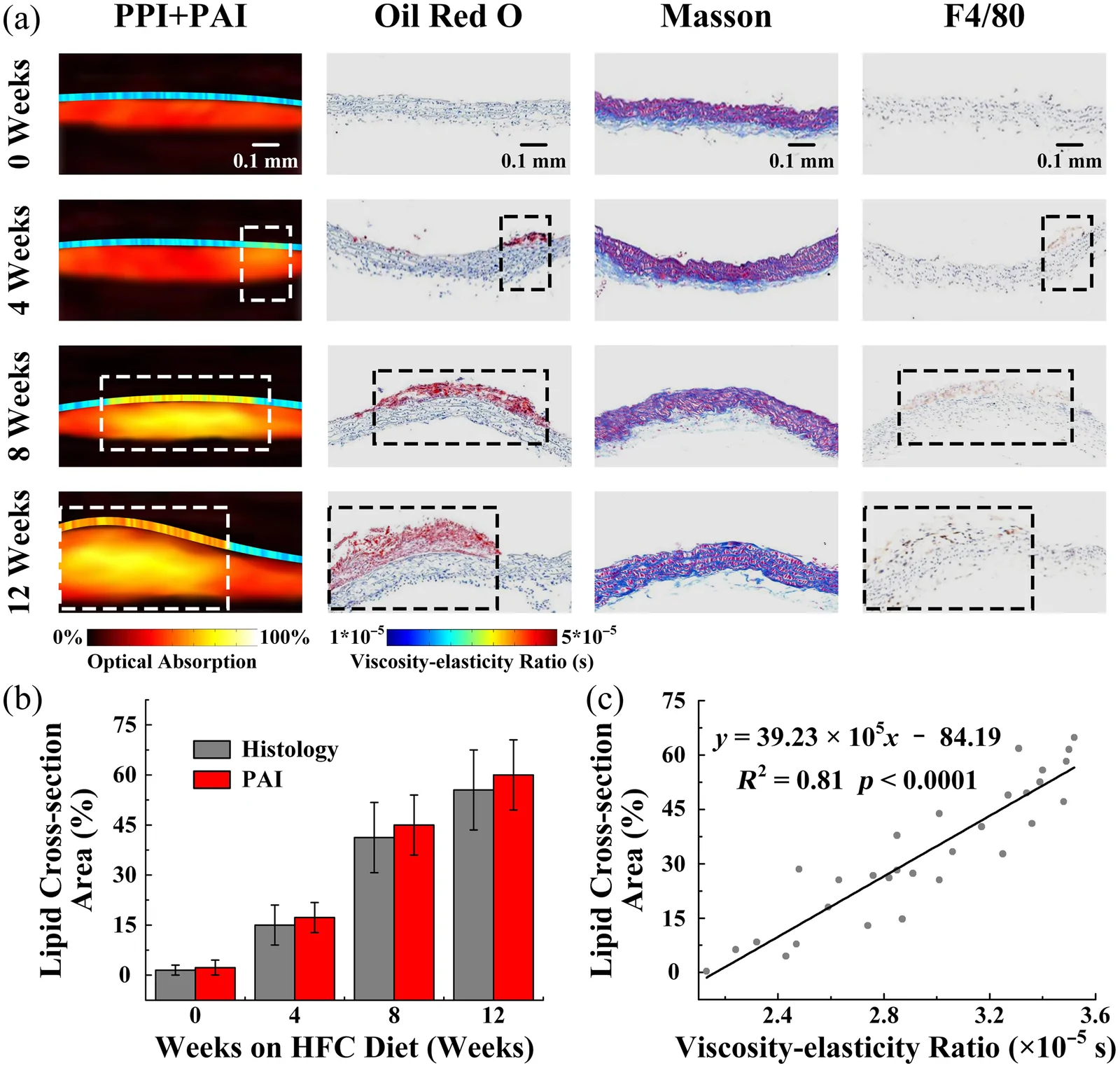
Lipid plaques characterized by PPI-PAI system. (a) Cross-section images of PPI + PAI, and histology (Oil Red O, Masson and F4/80 staining). The area enclosed by the dotted line indicates the presence of plaques. (b) Lipid cross-section area, measured by PAI and histology, was plotted against the duration of HFC diet. (c) High positive correlation between VER and lipid cross-section area (r=0.91, 95% CI: 0.85 to 0.96, p<0.0001, n=30, detected by Oil Red O dyeing results).

### 3.4 Characterization of Fibrous Plaques

When atherosclerosis developed to the stage of fibrous plaques, which had more complex structures and compositions, resulting in a large change in the VER. The most characteristic feature was the fibrous cap, which was formed by smooth muscle cells that migrated to the intima, proliferate, and secrete extracellular matrix components such as collagen and elastin. The PPI-PAI system was able to reflect the changes in fibrous cap thickness and lipid core area. Figure 6(a) shows the cross-section PPI and PAI images found in mice aortas of 20 to 24 weeks old, as well as the histological results (Oil Red O, Masson, and F4/80 staining). During the stage when the fibrous cap began to form, the plaque is relatively hard. Plaques in the 20-week-old mice had an average VER of $1.81\times 10^{-5}\;s$, which interrupted the trend of continuously rising VER values and was even lower than that of normal vessels ($\text{mean value}∼2.02\times 10^{-5}\;s$). At 24 weeks of HFC diet, when large numbers of macrophages began to attack the fibrous cap occurred in a subset of plaques, lesions became destabilized, and the VER values of thin-cap fibrous plaques reached its maximum ($3.77\times 10^{-5}∼4.68\times 10^{-5}\;s$). However, throughout the pathological progression, the area of the lipid core continued to increase. Figure 6(b) shows the lipid area, measured by PAI and histology, plotted against the duration of the HFC diet. In Fig. 6(c), the cap thickness ranged from 50  μm ($\mathrm{VER}∼4.55\times 10^{-5}\;s$) to 285  μm ($\mathrm{VER}∼1.26\times 10^{-5}\;s$). Linear regression analysis demonstrated a high negative correlation between cap thickness and VER at the plaque lesion level, with a slope of $-51.25\times 10^{5}\;\mu m/s$ (95% CI: $-57.47\times 10^{5}$ to $-45.62\times 10^{5}\;\mu m/s$), intercept of 323.33  μm (95% CI: 297.18 to 351.35  μm), $R^{2}=0.71$, p<0.0001, based on 30 plaque sections (n=30) obtained from six mice fed a HFC diet for 20 to 24 weeks. These data suggested that the fibrous cap thickness (essentially, it was the change in collagen fiber content) directly affected the VER of plaques.

**Fig. 6:**
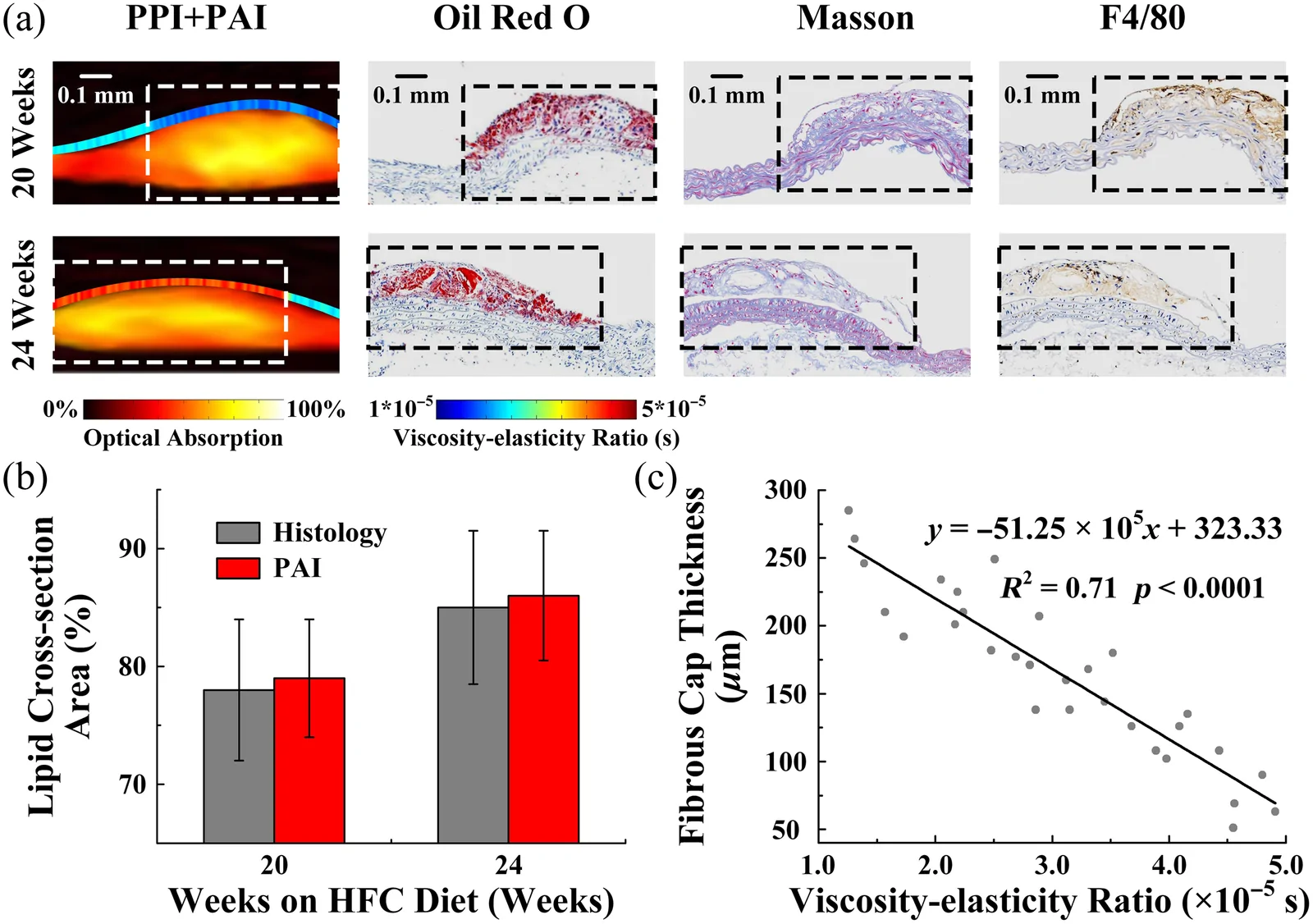
Fibrous plaques characterized by PPI-PAI system. (a) Cross-section images of PPI + PAI, and histology (Oil Red O, Masson and F4/80 staining). The area enclosed by the dotted line indicates the presence of plaques. (b) Lipid cross-section area, measured by PAI and histology, was plotted against the duration of HFC diet. (c) High negative correlation between VER and cross-sectional fibrous cap thickness (r=−0.85, 95% CI: −0.92 to −0.73, p<0.0001, n=30, detected by Masson dyeing results).

### 3.5 Correlation between VER and Inflammation Response in Plaques

To characterize the relationship between VER and inflammation cells, the distribution of VER and the macrophage content in plaques at different periods are shown in Figs. 7(a) and 7(b), respectively. The VER increased in the first four stages (lipid plaques), and mutations occurred in the last two stages (fibrous plaques) [Fig. 7(a)]. However, when plaque was in the stage of lipid plaque or fibrous plaque, the same VER might exist. PAI could be used to assist the judgment at this time. In Fig. 7(b), the macrophage content had steadily increased. At 24 weeks, the macrophage content reached the maximum (mean value∼34.12%), and the VER also reached its maximum ($\text{mean value}∼4.16\times 10^{-5}\;s$), at which time some plaques were in the thin-cap fibrous plaque stage. The statistical results [Fig. 7(c)] showed that during the lipid plaque stage (0 to 12 weeks of HFC diet), the VER value increased as the number of macrophages rose at the plaque lesion level, with a slope of $9.71\times 10^{5}\%/s$ (95% CI: $7.63\times 10^{5}\%/s$ to $11.78\times 10^{5}\%/s$), intercept of −17.02% (95% CI: −22.87% to −11.18%), $R^{2}=0.75$, p<0.0001, based on 30 plaque sections (n=30) obtained from 12 mice. During the stage when fibrous cap was present (20 to 24 weeks of HFC diet), the VER value increased as the number of macrophages rose at the plaque lesion level [Fig. 7(d)], with a slope of $1.88\times 10^{5}\%/s$ (95% CI: $0.30\times 10^{5}\%/s$ to $3.45\times 10^{5}\%/s$), intercept of 20.91% (95% CI: 15.93% to 25.89%), $R^{2}=0.12$, p=0.02, based on 30 plaque sections (n=30) obtained from six mice. The Bland–Altman agreement analysis based on 20 paired VER datasets was performed to quantify consistency between PPI (VER values after system and phantom calibration) and rheometer measurements [Fig. 7(e)]. The mean bias reached −0.046 (standard deviation of differences = 0.376), with 95% limits of agreement ranging from −0.783 to 0.692. The 95% CI of mean bias (−0.222, 0.130) contained zero, revealing no significant fixed systematic bias. Linear regression for proportional bias yielded a slope of 0.0436 (p=0.648), indicating that measurement discrepancies did not vary systematically with VER magnitude. Collectively, these findings demonstrated satisfactory quantitative consistency between the two detection modalities. All the results indicated that VER could accurately characterize the macrophage content only during the lipid plaque stage.

**Fig. 7:**
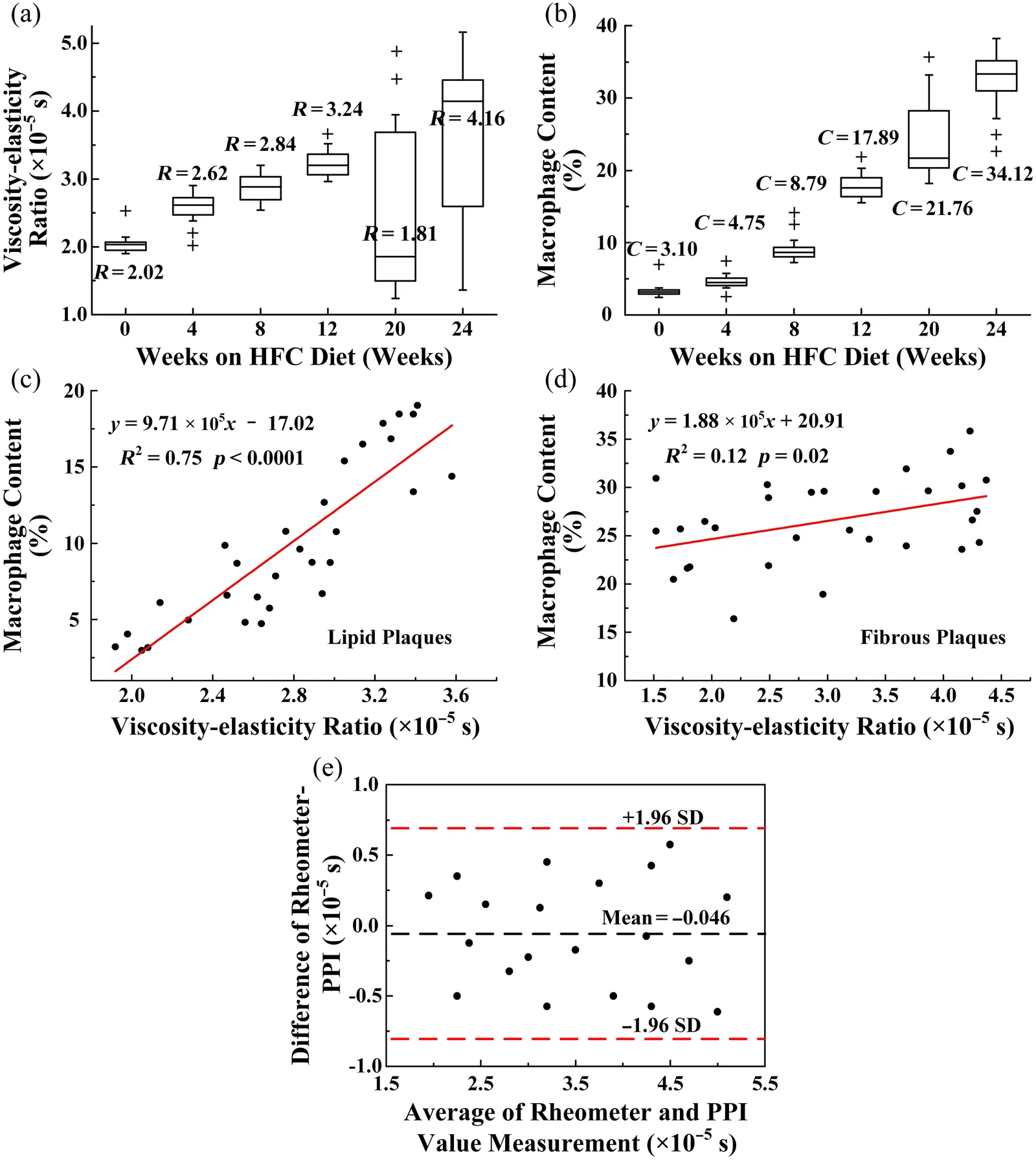
Correlation between VER and inflammation response in plaques. (a) VER and (b) Macrophage content of plaques fed with HFC diet for different times. Boxes have lines at lower, median, and upper quartile values. Whiskers show extent of remaining data. High positive correlation is demonstrated between VER and macrophage content in (c) cross-sectional lipid plaques (r=0.87, 95% CI: 0.75 to 0.94, p<0.0001, n=30), and (d) cross-sectional fibrous plaques (r=0.38, 95% CI: 0.07 to 0.64, p=0.02, n=30). (e) Bland-Altman tests for the cross-sectional plaque results between PPI and rheometer (n=20). C, macrophage content; R, viscosity-elasticity ratio; SD, standard deviation.

### 3.6 Effective Differentiation of Plaque Types by PPI-PAI Imaging System

When lipid plaques and fibrous plaques had the same VER value, the lipid content detected by PAI could help PPI to more accurately determine the lesion stage of plaques because the area of lipid core continued to increase as the plaque progresses pathologically. Figures 8(a) and 8(b) show cross-section images of PPI and PAI for lipid plaque (8 weeks of HFC diet) and fibrous plaque (20 weeks of HFC diet), respectively. Corresponding histological results (dotted box section), obtained by Oil Red O and Masson staining, are shown in Figs. 8(c) and 8(d), respectively. There had less lipid content in the lipid plaque, which was reflected in the smaller lipid cross-section area. By contrast, in the fibrous plaque, although the lipid content was more, VER had the same value as the stage of lipid plaque due to the presence of fibrous cap. Figures 8(e) and 8(f) show the distribution of VER and lipid content of plaques at two different stages, respectively. The VER of lipid and fibrous plaque was similar, at $∼2.91\times 10^{-5}\;s$. The lipid content of fibrous plaque was approximately three times that of lipid plaque. The experiment showed that the combination of PPI and PAI could provide a more accurate judgment for the assessment of plaque types.

**Fig. 8:**
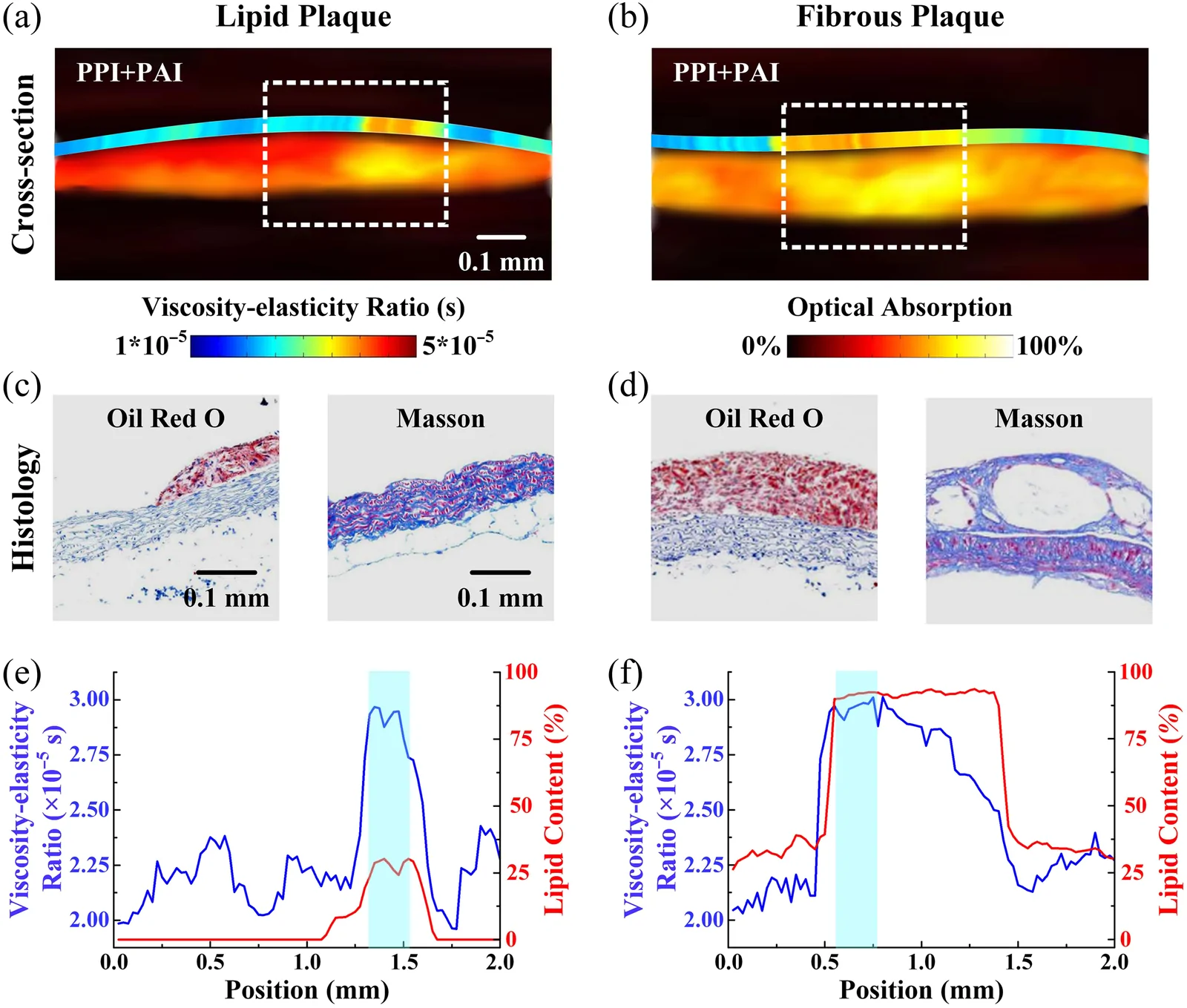
Characterization of lipid and fibrous plaque by PPI-PAI imaging system. (a) and (b) Cross-section images of PPI and PAI of lipid and fibrous plaque, respectively. (c) and (d) Corresponding histology by Oil Red O and Masson staining. (e) and (f) VER and lipid content distribution of plaque at two different stages, respectively.

**Table 1:** VER value and lipid core relative area for different types of plaques.

Plaque types	VER ($\times 10^{-5}\;s$)	Lipid core relative area (%)
Lipid plaques (n=27)	2.12∼3.59	7.46∼66.43
Thick-cap fibrous plaques (n=26)	1.14∼2.95	50.26∼75.84
Thin-cap fibrous plaques (n=4)	3.77∼4.68	70.32∼90.51

VER, viscosity-elasticity ratio.

The VER values and lipid core relative areas (LCRA) for different plaque types are shown in Table 1. The VER values ($3.77\times 10^{-5}∼4.68\times 10^{-5}\;s$) for thin-cap fibrous plaques were significantly higher (although there was a slight overlap in the LCRA values), making it easy to distinguish them from other plaque types. By contrast, there was some overlap in the VER and LCRA values between lipid plaques and thick-cap fibrous plaques. Therefore, ROC analyses were performed to compare the discriminative performance of single-feature and combined-feature models for distinguishing lipid plaques from thick-cap fibrous plaques, with all model outputs evaluated via leave-one-out cross-validation (Fig. 9). For PPI alone [Fig. 9(a)], the ROC analysis yielded an AUC of 0.86 (95% CI: 0.76 to 0.96). For PAI alone [Fig. 9(b)], the AUC was 0.82 (95% CI: 0.70 to 0.94). When PPI and PAI were combined [Fig. 9(c)], the AUC increased to 0.96 (95% CI: 0.91 to 1.00), indicating superior discriminative performance compared with either modality alone. The optimal cut-off threshold for the combined model, determined by maximizing the Youden’s index, was $\mathrm{VER}=2.53\times 10^{-5}\;s$ and LCRA=60.38%, yielding a sensitivity of 92.59% and a specificity of 88.46%. The corresponding confusion matrix results for PPI-only, PAI-only, and combined PPI-PAI analyses were provided in Part S7 of the Supplementary Material. These results demonstrated that the integration of PPI and PAI contrasts provided complementary information and substantially improved the diagnostic accuracy for distinguishing lipid plaques from thick-cap fibrous plaques.

**Fig. 9:**
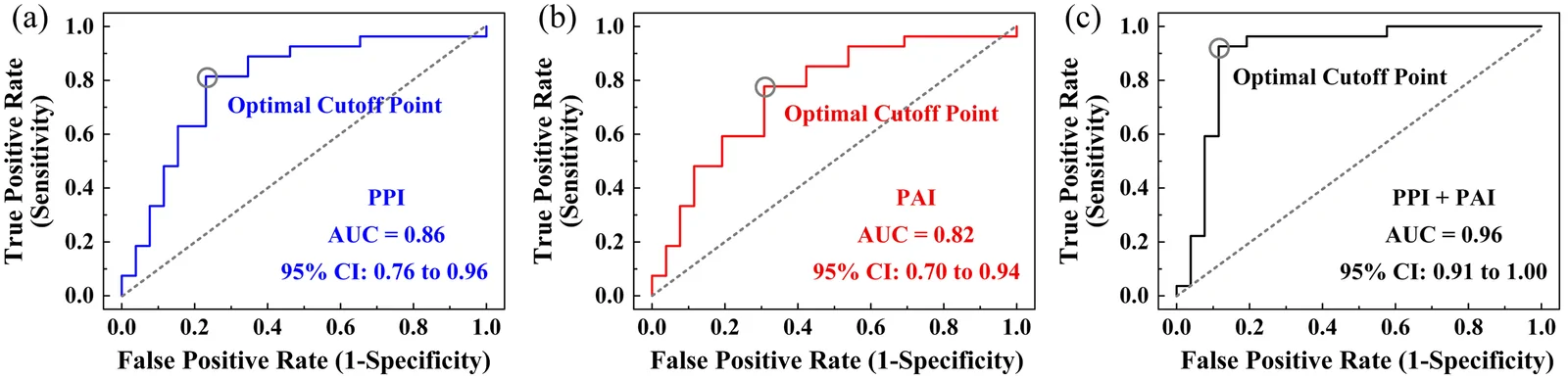
Performance characteristics of PA technique for binary discrimination between lipid plaques and thick-cap fibrous plaques. ROC curves obtained from leave-one-out cross-validation, illustrating classification performance using (a) PPI alone, (b) PAI alone, and (c) combined PPI-PAI features. The optimal cutoff point determined by maximizing Youden’s index is marked on the ROC curves. AUC values and their corresponding 95% CI are displayed within each panel.

## 4 Discussion

We have described PPI, a technique that measures PA phase of plaque molecules to provide an index of viscoelasticity. By analyzing the phase delay of PA signals to calculate the VER, and the experiments in ApoE mice show that the VER of atherosclerotic plaques depends on the plaque composition. In addition, the validation results of rheometer demonstrate the accuracy of PPI technique for VER detection. However, it is important to acknowledge that conventional rheometer (0.1 to 100 Hz) and PPI (50 kHz) probe fundamentally different frequency regimes, making direct comparison of VER values physically inappropriate. Nevertheless, the relative viscoelastic trends across phantoms were consistent between the two methods, and an empirical calibration curve was established to enable cross-modality interpretation. The 1 Hz reference frequency was chosen for rheometer data presentation as it corresponds to the physiological arterial pulsation rate. Importantly, the relative ranking of VER across plaques remained unchanged across the entire 0.1 to 100 Hz sweep, confirming that frequency disparity does not compromise tissue differentiation. Thus, although VER values are not directly interchangeable, the calibration and consistent trends validate PPI for comparative viscoelastic characterization. Furthermore, the intensity information of the PA signal is also analyzed to achieve the specific identification of lipids. The combination of PPI and PAI enables the simultaneous characterization of the viscoelastic properties and lipid distribution of plaques, thus achieving effective differentiation of plaque types. Compared with previously proposed multimodal systems (incorporating optical coherence tomography, photoacoustic, and viscoelastic imaging),^29^ this study demonstrates that VER, as a direct mechanical parameter, sensitively detects plaque compositional changes. Moreover, by exploiting only photoacoustic phase (VER) and intensity (lipid composition), the PPI-PAI approach achieves diagnostic performance comparable to sophisticated multimodal systems, simplifying hardware and enhancing clinical translation potential. This constitutes a pivotal advance from conventional multiparameter characterization toward effective plaque characterization integrating VER and lipid composition.

One aim of the present study is to demonstrate that VER values derived from PPI can identify thin-cap fibrous plaques, with values approximating $3.77\times 10^{-5}∼4.68\times 10^{-5}\;s$ and being markedly higher than those of other plaque types. It is particularly important to note that we cannot simply define the thin-cap fibrous plaques (cap thickness<65  μm) as vulnerable plaques because a fibrous cap thickness of less than 65  μm is considered one of the key parameters for assessing human vulnerable plaques, rather than a result specific to mice aortic plaques. Therefore, the experimental results do not provide diagnostic thresholds (or detection ranges) for the VER of vulnerable plaques. Another aim of the study is to explore the relationship between VER and the main components of plaque. The detection of VER show the high positive correlation between VER and lipid cross-section area ($R^{2}=0.81$, p<0.0001) and macrophage content ($R^{2}=0.75$, p<0.0001) in lipid plaque lesions. Likewise, in fibrous plaques, a strong negative correlation ($R^{2}=0.71$, p<0.0001) is demonstrated between VER and fibrous cap thickness, and a high positive correlation ($R^{2}=0.12$, p=0.02) is demonstrated between VER and macrophage content. Plaque features such as lipid core area, fibrous cap thickness, and macrophage content are interrelated and contribute to the viscoelastic properties of plaques. The significant linear correlation does imply that the measures are highly related. The last aim of the study is to verify whether PPI-PAI can achieve effective differentiation of plaque types. The considerable overlap in VER and LCRA distributions exists between lipid plaques and thick-cap fibrous plaques. The combined PPI-PAI classifier achieves a substantially higher AUC (0.96) than either individual modality (PPI, 0.86; PAI, 0.82). This observation underscores the complementary nature of the viscoelastic (PPI) and compositional (PAI) contrasts and suggests that their joint application effectively enhances diagnostic performance in discriminating plaque types.

This study also has some limitations. In terms of system imaging, first, the atherosclerotic specimens, used in the *ex vivo* study, are stored at 4°C in PBS before imaging; it is possible that minor degradation of the specimens may have occurred. Hence, it is possible that quantitative measurement of VER may slightly vary under *in vivo* conditions. However, in this study, because the VER differences among the different constituent plaque types are highly statistically significant, we predict that these relative differences would also exist under *in vivo* conditions. Second, lock-in detection takes a considerable amount of time (point-by-point scanning an ∼1  cm×1  cm area with a 25  μm step size takes about 2 h). It is inadequate for real-time imaging in clinical application. In future research, we can improve the signal-to-noise ratio of the PA signal, employ cross-correlation algorithms between the PA signal and the reference signal that does not require the lock-in detector device,^30^ or use a microlens array to achieve multichannel operation,^31^ to increase the data-acquisition speed. Last, in PPI, the VER values are computed over the entire PA signals and the reference signals in the lock-in detector. As a result, the effect of viscoelasticity is integrated over the illuminated volume in the atherosclerotic plaque, and depth-resolved spatial information is lost. To overcome this problem, an electrically tunable lens will be used in the future to realize a fast variable-focus PPI with a large range of imaging depth, which may be possible to obtain depth information of plaque VER distributions. In addition, optical coherence tomography can be used to achieve the depth resolution of the PA’s mechanical response.^32^^,^^33^

In terms of statistics and research design, the cross-sectional design cannot capture temporal plaque progression within individual animals. Although plaque typing was performed blindly using predefined histopathological criteria, partial confounding persists between plaque type and HFC diet duration. Because multiple cross-sections per mouse yield clustered, nonindependent measurements, the reported statistics reflect plaque level associations and are not generalizable to animal level replicates. Hierarchical mixed effects modelling to correct intra-animal clustering was infeasible with only three mice per time point (insufficient for stable convergence), whereas animal level averaging would erase intra-lesion heterogeneity and mask the structure–function relationships of interest. Thus, with limited animal level power, apparent significance may stem from abundant sectional data rather than genuine biological effects, and we refrain from causal inferences. Larger cohorts with longitudinal or lineage tracing designs are needed to validate these lesion level findings.

Further limitations include the fact that the present study is a preliminary *ex vivo* investigation based on a modest number of preselected plaque samples, which may not fully recapitulate the histopathological heterogeneity inherent to human atherosclerotic lesions. Accordingly, although the combined PPI-PAI approach demonstrates promising classification performance, these findings warrant further validation in larger and more diverse cohorts prior to clinical translation. From a translational perspective, the current setup cannot assess plaque vulnerability or predict clinical events; the reported classification outcomes remain preliminary and should not be interpreted as evidence of high diagnostic accuracy. Future investigations should therefore adopt a blinded diagnostic design with prespecified histological definitions of plaque vulnerability, a separate training set for threshold selection, and an independent test set. Only with such supporting evidence can the clinical utility of PPI-PAI be determined. Furthermore, given the intrinsic compatibility of PPI-PAI principles with intravascular catheters, the development of catheter-integrated dual-modality systems represents a necessary next step to enable *in vivo* plaque evaluation, which is critical for unlocking the clinical value of this imaging strategy.

## 5 Conclusion

In summary, we have introduced PPI as a quantitative method for characterizing the component-dependent VER of atherosclerotic plaques. PPI enables identification of thin-cap fibrous plaques and measures an index of viscoelasticity that is related to plaque lipid core area, fibrous cap thickness, and macrophage content. The combination of PPI and PAI permits discrimination among different plaque types. Given the wealth of information provided by the PA technique and its potential for intracoronary use, we anticipate that PPI-PAI will prove highly useful for the evaluation of coronary atherosclerotic lesions.

## Supplementary Material

10.1117/1.JBO.32.S1.S14904.s01

## Data Availability

The original contributions presented in the study are included in the article, and further inquiries can be directed to the corresponding author.
