# Supplementary material for: Photoacoustic phase and intensity dual-modality imaging for characterization of plaque viscoelasticity and lipid composition: a feasibility study
Source: J Biomed Opt. 2026 Oct 28;32(Suppl 1):S14904. doi: 10.1117/1.JBO.32.S1.S14904 (PMC13714978; doi:10.1117/1.JBO.32.S1.S14904)
Supplement: Supplementary file 1 [file JBO_032_S14904_SD001.pdf]

Supplementary Materials

Photoacoustic phase and intensity dual-modality imaging for characterization of plaque viscoelasticity and lipid composition: A feasibility study

Yuxuan Liang,[a] Yiting Tan,[a] Ao Jiang,[a] Heng Zhang,[a] and Pingping Wang[a, b, *]

S1 Phase calibration workflows

1 Subtraction of intrinsic system delay

1.1 Reference experiment for system baseline phase

All fixed phase offsets originating from electronic circuits and optical transmission paths were quantified through a dedicated reference calibration experiment. An ultrathin carbon nanotube film (< 20 nm) was uniformly deposited on a glass coverslip at the imaging focal plane. Upon modulated laser irradiation, the carbon film generated instantaneous thermoelastic expansion with negligible viscoelastic retardation. The phase reading acquired from this reference calibration was defined as the system baseline $\boldsymbol{\delta_0} \approx$ **(3.43 ± 0.13)°** (a total of 10 measurements; the data are shown in Table S1), which was subtracted from all raw phase data collected during subsequent vascular plaque imaging. The coefficient of variation (CV) was determined to be 3.79%, indicating that the measurement data exhibited satisfactory repeatability and stability.

Table S1 System baseline phase measurement.

**Times**	**1**	**2**	**3**	**4**	**5**	**6**	**7**	**8**	**9**	**10**	**CV**
$\boldsymbol{\delta_0}$ **(°)**	3.28	3.57	3.41	3.55	3.44	3.59	3.32	3.19	3.47	3.50	3.79%

CV, coefficient of variation.

1.2 Laser-induced phase effects and calibration

The 1064 nm quasi-continuous laser (DS20HE-1064D/R, PHOTONICS) delivered a train of 22 ns pulses at a repetition rate of 50 kHz for periodic photoacoustic (PA) excitation. This pulsed source introduced a stationary phase offset originating from the inherent timing delay between the internal trigger and optical pulse output, as well as minor pulse-to-pulse timing jitter that contributed to phase noise. Slow drift in pulse energy or beam profile could further perturb the spatial optical deposition within the 0-100 μm focal layer and induce secondary phase deviations [S1, S2]. Beneficially, the 22 ns

short pulse duration satisfied the thermal confinement criterion [S3], so thermally induced phase lag was negligible. To mitigate laser-related phase artifacts, baseline calibration using an ultrathin carbon nanotube film reference absorber was performed prior to tissue measurement to eliminate the stationary laser-associated phase bias. The 50 kHz synchronous trigger signal output from the laser was fed directly into the lock-in amplifier as the reference clock to suppress phase fluctuations caused by pulse timing jitter. Rigid optical alignment and continuous optical power monitoring were maintained throughout experiments.

1.3 Calibration of the transducer, preamplifier and lock-in amplifier

Beyond static system phase baseline subtraction via the carbon nanotube film glass coverslip, fixed single-frequency phase offsets of the ultrasonic transducer, preamplifier and lock-in amplifier at 50 kHz were separately quantified and compensated via three independent targeted calibration measurements, which complemented the static baseline correction to eliminate all hardware-induced phase artifacts under the experimental working condition.

(1) Calibration for the ultrasonic transducer

The positions of the two transducers were fixed with a rigid holder throughout all measurements, and the source–detector distance was kept constant. Therefore, we only needed to consider the 50 kHz ultrasonic transducer used for photoacoustic phase imaging (PPI). Piezoelectric pulse-echo measurement was performed exclusively at the fixed working frequency of 50 kHz to quantify the intrinsic phase lag of the transducer piezoelectric chip. A planar high-impedance acoustic reflector was positioned at a constant, precisely known distance from the transducer (~2.0 cm) within 0.9% normal saline. Short electrical excitation pulses were delivered to the piezoelectric wafer, and the reflected echo signal was recorded at this single frequency. The phase lag between the transmitted excitation pulse and received echo was extracted as the constant transducer phase offset [$\boldsymbol{\delta_{UT}} \approx$ **(2.61 ± 0.06)°**, a total of 10 measurements; the data are shown in Table S2**]** for subsequent phase compensation. The CV was 2.30%, confirming the robustness and stability of the measurement data.

Table S2 Piezoelectric pulse-echo phase offset measurement.

**Times**	**1**	**2**	**3**	**4**	**5**	**6**	**7**	**8**	**9**	**10**	**CV**
$\boldsymbol{\delta_{UT}}$ **(°)**	2.66	2.71	2.62	2.54	2.59	2.55	2.63	2.57	2.69	2.51	2.30%

CV, coefficient of variation.

(2) Calibration for the preamplifier

Before each measurement, the phase transfer function of the preamplifier was characterized using a reference electrical signal with well-defined phase. The frequency-dependent phase offset introduced by the preamplifier was subsequently quantified and subtracted from the raw phase data acquired from tissue samples. After this calibration procedure, the residual static phase contributed by the preamplifier was less than 0.5°. To further suppress frequency-dependent phase distortion, the preamplifier was selected with a bandwidth exceeding ten times the maximum modulation frequency employed in the PPI measurement. Meanwhile, the alternating current (AC)-coupling cutoff frequency of the amplifier was configured to be lower than 0.1 times the minimum modulation frequency, ensuring minimal phase roll-off across the entire working frequency range and preserving the fidelity of the viscoelasticity-related phase information for viscosity-elasticity ratio (VER) quantification.

(3) Calibration for the lock-in amplifier

A precisely characterized resistor-capacitor (RC) network with tabulated fixed resistance and capacitance values acted as the standard phase reference. A 50 kHz sinusoidal signal matching the laser modulation waveform was fed into this RC circuit, and its theoretical phase shift was analytically calculated from circuit impedance parameters. The phase output measured by the lock-in amplifier was compared with this theoretical reference phase to quantify the amplifier's fixed demodulation phase error [$\boldsymbol{\delta_{LA}} \approx$ **(1.70 ± 0.03)°**], a total of 10 measurements; the data are shown in Table S3**]**. The CV of 1.76% was obtained, demonstrating a high degree of measurement consistency and reliability.

Table S3 Lock-in amplifier phase error measurement.

**Times**	**1**	**2**	**3**	**4**	**5**	**6**	**7**	**8**	**9**	**10**	**CV**
$\boldsymbol{\delta_{UT}}$ **(°)**	1.74	1.68	1.65	1.70	1.73	1.72	1.69	1.71	1.74	1.68	1.76%

CV, coefficient of variation.

By combining all of these corrections, the total system phase uncertainty after correction was reduced to less than 1°.

1.4 The effect of optical path length

Regarding the optical path length, we considered two contributions. First, the fixed optical path inside the optical train and the static ~3 cm working distance between the objective lens and the tissue surface introduced a constant propagation delay for all measurements and was inherently included in

the system baseline phase $\boldsymbol{\delta_0}$, which we already subtracted using the ultrathin carbon nanotube film reference. Second, the variable optical path originates from light penetration depth within the tissue, between the tissue surface and subsurface absorbing features. At the modulation frequency of 50 kHz, a 100 μm depth variation inside the tissue yields an additional phase shift of less than 6.0×10^{-6} degrees, which is many orders of magnitude below the phase noise floor. Therefore, optical path length variation does not introduce any meaningful bias into the measured phase, and no further correction is required beyond the baseline subtraction already performed (Table S1). It should be noted that the 100 μm tissue depth is selected because the laser focal depth within tissue is approximately 100 μm, and the acquired PA signal reflects the averaged VER across the depth range of 0 to 100 μm, which justifies using this value for phase error estimation.

2 Decoupling of acoustic propagation delay from tissue thickness

2.1 Acoustic propagation delay correction

Acoustic propagation delay arises from the time-of-flight (ToF) of PA signals propagating from tissue optical absorbers to the ultrasonic transducer, which generates a distance-dependent phase lag defined as $\boldsymbol{\delta_{AP}} = 2\pi fd/c$, where d denotes the acoustic propagation path length and c represents the sound speed of the medium [S4, S5]. In the present microscopic setup, the acoustic separation between the ultrasonic transducer and the laser focal plane is fixed at approximately 2 cm (keep conditions consistent for each experiment). At the modulation frequency of 50 kHz with 0.9% normal saline as the coupling medium (c ≈ 1530 m/s) [S6], this fixed distance corresponds to a constant phase offset of approximately 235.3°, which can be quantified and subtracted as a stationary baseline term during post-processing.

2.2 Tissue thickness and boundary reflection management

Given that PPI interrogated only the superficial tissue layer (0-100 μm) defined by the optical focal depth, the influence of tissue thickness variation on phase measurement was inherently limited. Within this shallow detection window, the maximum acoustic propagation path-length variation across the imaged layer was 100 μm, corresponding to a phase error of approximately 1.18° at the modulation frequency of 50 kHz (calculated as $2\pi \times 50\times10^3 \times 100\times10^{-6} / 1530 \approx 0.0205$ rad ≈ 1.18°). Such phase variation should be considered and compensated in subsequent phase quantification. Furthermore, because the PA signals originated exclusively from this superficial region, reflections from deep plaque boundaries or the vessel adventitia did not reach the detection window and thus did not contribute to

the measured phase. Consequently, tissue thickness variation did not introduce significant phase artifacts in the current configuration. Nevertheless, for future applications targeting deeper lesions where such effects may become relevant, we acknowledge that time-gating or path-length correction strategies would be necessary [S7, S8].

3 Influences of acoustic impedance mismatch and signal-to-noise ratio (SNR)

3.1 Phase effects of acoustic impedance mismatch

At the 50 kHz modulation frequency adopted in this work, phase deviations originating from acoustic impedance discontinuities represented stationary systematic offsets instead of stochastic noise components. With regard to the saline-vascular tissue boundary, 0.9% normal saline (~1.5 MRayl) and vascular soft tissue (1.5-1.7 MRayl) exhibited highly consistent acoustic impedance values [S9, S10], which yielded marginal interfacial impedance mismatch and thereby negligible phase distortion. As for the transducer-saline interface, the ultrasonic transducer was integrated with a dedicated acoustic matching layer to mitigate the massive impedance disparity between piezoelectric ceramics (~30 MRayl) and 0.9% normal saline (~1.5 MRayl) [S9, S10]. Only minor static phase bias persisted after such impedance matching. Critically, phase artifacts derived from both interfaces were purely systematic and invariant under steady experimental setups; they remained independent of intrinsic tissue viscoelastic features and spatial measurement positions across the field of view. We had implemented a comprehensive system phase calibration protocol, which reduced the total system phase uncertainty to less than 1° and effectively eliminated all systematic phase deviations, including those arising from acoustic impedance mismatch.

3.2 The effect of SNR on phase measurement

Comprehensive quantitative characterization of SNR was implemented across all imaging pixels to guarantee reliable viscoelastic quantification. A lock-in amplifier with a time constant of 30 ms was employed, which provided a phase noise floor of approximately 0.5° under the experimental conditions (defining the minimum resolvable random phase fluctuation of the hardware). To eliminate unreliable low-quality data prone to severe phase distortion, a hard threshold of SNR > 15 dB was imposed; only pixels satisfying this criterion were retained for reconstructing the final VER parametric maps. The above SNR screening and noise control strategy ensured the statistical validity and precision of subsequent VER quantification.

4 The effect of coupling medium

The 0.9% normal saline served as the acoustic coupling medium. At the modulation frequency of 50 kHz, this medium exhibits negligible acoustic dispersion and a well-defined sound speed of approximately 1530 m/s [S6]. It introduces a stationary acoustic propagation phase offset determined by the fixed acoustic path between the PA source and the ultrasonic transducer, which can be fully compensated during system calibration. Potential phase perturbations arising from microbubbles or temperature fluctuations were minimized by degassing the saline solution and maintaining stable temperature throughout measurements. The saline exhibits negligible optical absorption at the working wavelength and does not generate interfering PA signals.

5 The effect of optical absorption and thermal-generation characteristics

Spatially heterogeneous optical absorption within the 0-100 μm laser focal layer leads to distributed PA sources at different depths [S11]. PA signals generated from varying depths travel different acoustic distances to the ultrasonic transducer, yielding integrated phase deviations superimposed on the target viscoelastic VER phase. Additionally, finite thermal relaxation and thermal diffusion can introduce extra thermal-related phase lag if the thermal confinement condition is not satisfied, which cannot be separated mechanically from the measured phase [S12, S13]. Nonlinear optical absorption under excessive laser fluence may further distort waveform and induce phase distortion [S14, S15]. In PPI-photoacoustic absorption imaging (PAI) system, the 22 ns short pulse of the 1064 nm quasi-continuous laser fulfills the thermal confinement criterion, which largely suppresses thermally induced phase lag. The baseline calibration using the ultrathin carbon nanotube film reference absorber is applied to compensate for the integrated phase bias originating from uneven light deposition. Laser fluence is maintained within the linear PA response regime to avoid nonlinear signal distortion.

6 Calibration procedure using materials with known viscoelastic properties

A series of agar-gelatin phantoms with varying agar concentrations (1.0%, 1.5%, 2.0%, 2.5%, and 3.0%) was prepared (prepare 5 samples of each concentration). Ager-gelatin and ink concentrations (0.5%) were kept constant across all phantoms to ensure uniform background viscoelasticity and optical absorption. The phantoms were analyzed using the PPI, and the VER values were recorded after calibrating the phase delay of the PA signal. Subsequently, the viscoelastic properties of these phantoms (the same phantoms) were independently characterized using the rheometer (Anton Paar MCR 302e) under dynamic oscillation mode at a frequency range 0.1-100 Hz (the 1 Hz frequency serves as a

consistent reference for empirical calibration with PPI measurements at 50 kHz) and at physiological temperature (37 °C). The true VER of each phantom was then calculated, which represented the ratio of the viscosity coefficient to the Young's modulus. A calibration curve was established by plotting the PA-derived VER against the rheometer-measured VER and performing linear regression analysis. In Fig. S1, results revealed a significant linear association between PPI-detected VER and rheometer-detected VER, with a slope of 2.69 (95% CI: 2.62 to 2.77), intercept of 0.17×10^{-6} s (95% CI: 0.15×10^{-6} s to 0.20×10^{-6} s), $R^2 = 0.90$, $p < 0.0001$, n = 25. This calibration equation ($VER_R = 2.69VER_{PA} + 0.17 \times 10^{-6}$) was then applied to correct all subsequent tissue measurements.

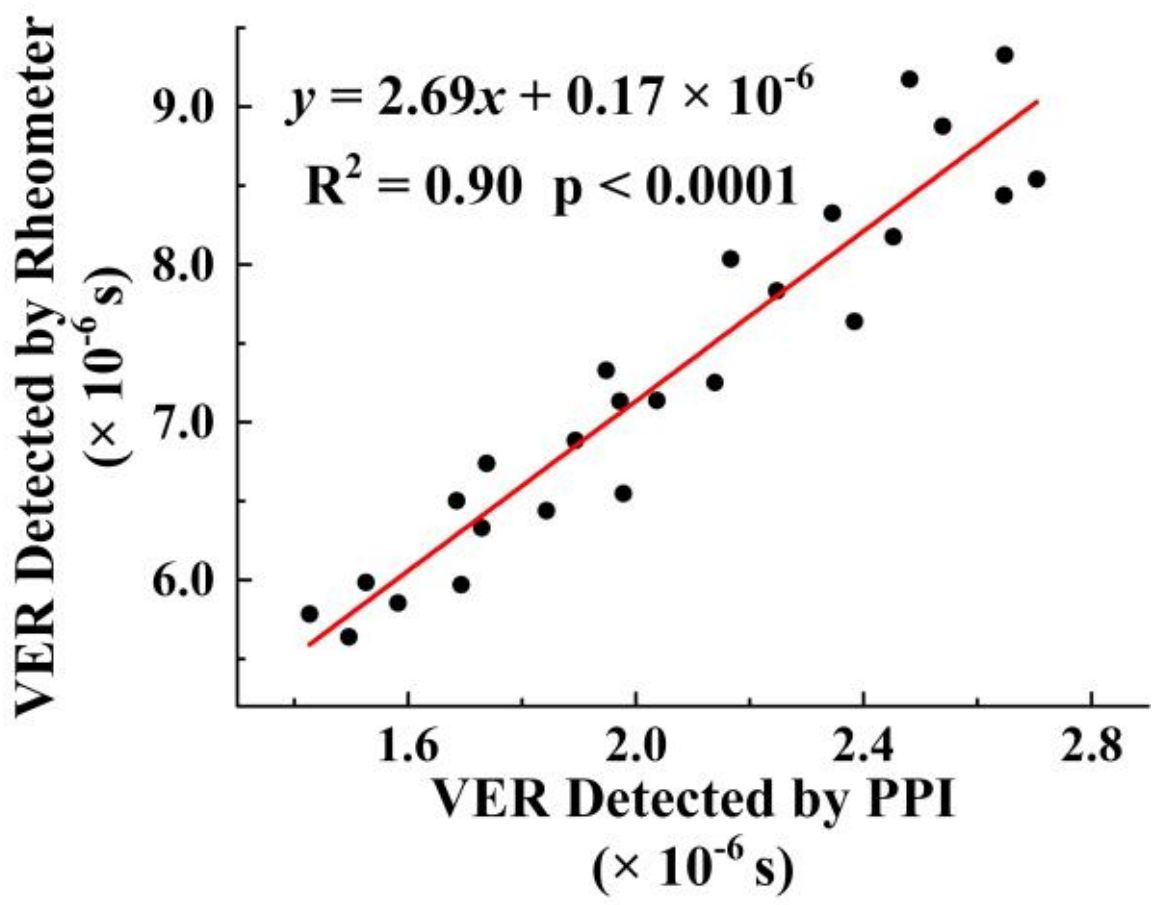

Fig. S1 The calibration curve between the PA-derived VER against the rheometer-measured VER. r = 0.95, 95% CI: 0.93 to 0.98, $p < 0.0001$, n = 25.

After sequential removal of optical, acoustic, instrumental, positional, coupling medium, thermal-generation characteristics, and noise-related phase artifacts, and validated by calibrated viscoelastic reference phantoms, the differential phase signal extracted from plaque tissue can be reliably attributed to intrinsic tissue viscoelasticity governed by the Kelvin-Voigt constitutive relation. The reported VER parametric maps therefore reflect genuine tissue VER rather than measurement artifacts. The Bland-Altman agreement analysis based on 20 paired VER datasets was performed to quantify consistency between PPI (VER values after system and phantom calibration) and rheometer measurements [Fig. 7(e) in the manuscript]. The mean bias reached -0.046 (standard deviation of differences = 0.376), with 95% limits of agreement ranging from -0.783 to 0.692. The 95% CI of mean bias (-0.222, 0.130) contained zero, revealing no significant fixed systematic bias. Linear regression for proportional bias yielded a slope of 0.0436 (p = 0.648), indicating measurement discrepancies did not vary systematically with VER magnitude. Collectively, these findings demonstrated satisfactory

quantitative consistency between the two detection modalities.

S2 Rheometer parameter settings

All rheological measurements were performed as shown in Table S4.

Table S4. Rheometer (Anton Paar MCR 302e) test parameters and instructions.

Parameter	Value/Description
**Sample size**	n = 25 independent phantoms (5 different agar-gelatin concentrations, with 5 samples for each concentration); and n = 20 independent mice aortic plaques.
**Geometry**	Parallel plate, 8 mm diameter (phantoms) and 1 mm diameter (tissue, due to limited size). Gap height: ~ 1 mm for phantoms, ~ 0.5 mm for tissue.
**Loading mode**	Dynamic oscillatory shear (controlled stress).
**Frequency range**	0.1 Hz to 100 Hz (frequency sweep) for each sample, primary comparison at 1 Hz (physiological cardiac frequency).
**Strain amplitude**	1% (within the linear viscoelastic regime, confirmed by amplitude sweep).
**Temperature**	37.0 °C (physiological), maintained by a Peltier plate and verified before each measurement.
**Hydration state**	Samples were kept in phosphate-buffered saline during testing. A humidity chamber was used to prevent drying. For tissue, the adventitia was gently blotted but kept moist.
**Tissue orientation**	Vascular samples were placed with the radial thickness direction of the vessel wall aligned parallel to the rheometer shear plane, to ensure that the VER values measured by PPI are comparable to those measured by the rheometer.

For rheological reference, 1 Hz was selected as the representative frequency for data presentation. This choice was physiologically motivated, as 1 Hz corresponded to the fundamental pulsatile frequency of the human arterial system, a condition routinely employed in plaque rheology studies to characterize the viscoelastic behavior of vascular tissues under physiological loading [S16]. This single-frequency comparison, however, was not intended to imply frequency equivalence between rheometer and PPI. Instead, it provided a consistent reference point for establishing an empirical linear calibration between VER values derived from the two modalities. Importantly, across the entire 0.1-100 Hz frequency sweep, both methods consistently ranked the viscoelastic contrast among different plaque types, confirming that the frequency mismatch did not compromise the reliability of tissue differentiation.

S3 PAI signal processing and lipid characterization

1 Laser-energy normalization and fluence correction

1.1 Pulse-to-pulse laser energy normalization

In the present system, each PA signal was normalized to the corresponding laser pulse energy to eliminate pulse-to-pulse energy fluctuations, with residual fluctuations maintained below 2% [S17, S18]. This normalization was essential because the PA signal amplitude was linearly proportional to the incident laser fluence; any pulse-to-pulse energy variation would otherwise have introduced spurious amplitude fluctuations that could have been misinterpreted as changes in optical absorption [S19, S20]. Specifically, at each excitation point, a reference optical signal that bypassed the sample was acquired synchronously using a fast photodiode placed in the beam path prior to the sample. This reference signal provided a real-time measure of the pulse energy for each laser shot. After averaging over 100 pulses, a widely adopted approach that improved the signal-to-noise ratio (SNR) by a factor of approximately $\sqrt{100} = 10$, the raw PA signal was divided by the corresponding averaged reference signal, thereby effectively suppressing energy drift and shot-to-shot variability [S21-S23].

1.2 Correction of spatial illumination inhomogeneity

In addition to pulse-to-pulse energy normalization, spatial inhomogeneity of the illumination beam could introduce significant systematic errors in quantitative PA imaging. Even when the total pulse energy was constant, a non-uniform beam profile resulted in varying local fluence across the imaging field of view, which in turn caused position-dependent variations in signal amplitude that were unrelated to tissue optical absorption [S24]. To address this issue, a beam profiler was employed to measure the spatial photon flux distribution across the sample surface. The measured beam profile was then used to generate a two-dimensional correction map, and a photon flux correction factor was applied to each imaging pixel to compensate for the spatial inhomogeneity of the illumination [S25]. This pixel-wise correction was critical for quantitative PA imaging, as it ensured that the detected signal amplitude accurately reflected the local optical absorption coefficient rather than variations in the incident light distribution. The combined use of pulse-to-pulse energy normalization and spatial fluence correction ensured that the measured signals were comparable across different imaging positions and experimental sessions, providing a robust foundation for subsequent quantitative analysis.

1.3 Depth-dependent fluence compensation

To account for depth-dependent photon flux attenuation arising from tissue absorption and

scattering, a layer-by-layer compensation algorithm based on the Beer-Lambert law was further implemented. In biological tissues, the effective optical attenuation coefficient, which depended on both the absorption and scattering properties of each layer, determined the rate at which the incident fluence decayed with depth [S26, S27]. This attenuation was wavelength-dependent and could introduce substantial depth-related biases into PA signal quantification, particularly when target structures were located at varying depths [S27]. In our implementation, the tissue was treated as a series of layers with different attenuation coefficients along the acoustic axis. The algorithm estimated the local fluence at each depth by iteratively accounting for the cumulative attenuation from all overlying layers, using the known or estimated optical properties of each layer. At each imaging pixel, the measured PA signal was divided by the corresponding estimated fluence to recover a signal proportional to the local optical absorption coefficient. This compensation was essential for quantitative PA imaging, as it mitigated the spectral coloring effect that could otherwise have distorted functional parameter estimates [S28]. Together with the energy normalization and spatial fluence correction described above, these procedures ensured the accuracy and reliability of the subsequent quantitative analysis.

2 Background subtraction and water/other absorber handling

The PA signal measured at 1700 nm contains contributions not only from lipid but also from water and other endogenous absorbers. At this wavelength, the first overtone of the C-H stretching vibration gives lipids a distinct absorption band, yet water also exhibits a certain level of comprehension in the 1700-1900 nm range, which can confound the quantification of lipid content in hydrated tissue environments [S29, S30]. Therefore, to isolate the lipid-specific component of the PA signal, it was necessary to characterize and subtract the background contribution arising from water and other non-lipid absorbers.

To this end, a background subtraction protocol was implemented using a water-only phantom composed of 2% agarose gel prepared with deionized water. The agarose concentration was chosen to match the mechanical properties of the tissue samples, while the use of deionized water ensured that the phantom's optical absorption was dominated by water, free from interfering chromophores. This phantom was imaged under the same experimental conditions as the tissue samples, including laser parameters and detection settings. The mean PA signal amplitude from a region of interest within the phantom was then used as an estimate of the water-induced background. This background was

subsequently subtracted from the tissue signals on a pixel-wise basis to yield a water-corrected PA signal.

Because water absorption at 1700 nm is significantly lower than the C-H absorption of lipids, the residual water contribution to the tissue signal after subtraction was expected to be minimal [S31]. To confirm the efficacy of this correction, the lipid-to-water signal ratio was compared before and after subtraction, revealing a significant enhancement in lipid contrast (approximately 30-40% enhancement). The water phantom data were also used to assess the contribution of the coupling medium (0.9% normal saline) to the overall background signal, confirming that the saline contribution was negligible under our experimental conditions. This background subtraction protocol, in conjunction with the energy normalization and fluence compensation procedures, ensures that the PA signal amplitude more accurately reflects lipid-specific absorption, thereby improving the reliability of quantitative lipid assessment in atherosclerotic plaques.

3 Segmentation threshold

To derive the quantitative lipid area, a segmentation threshold was applied to the normalized, background-subtracted PA images. The threshold was determined based on the image noise statistics [S32]. Specifically, a noise-only region devoid of any signal (within a water-only phantom) was identified, and its mean (μ = 1.24) and standard deviation (σ = 4.05) were calculated, with signal amplitudes expressed in Analog-to-Digital Converter (ADC) counts. The threshold was then defined as $T = \mu + k \cdot \sigma$, where k is a multiplicative factor. This approach ensures that only pixels with signal intensities significantly above the background noise are classified as lipid-positive, thereby minimizing false positives [S32, S33].

To validate the robustness of this threshold and to avoid bias from arbitrary parameter selection, a sensitivity analysis was conducted by systematically varying k from 2.0 to 5.0 in increments of 0.5 and calculating the corresponding lipid area at each level. The resulting lipid areas were plotted as a function of k to identify the range over which the quantitative measurements remained stable. As shown in Fig. S2(a), the lipid area exhibited a clear plateau region for k values between 2.5 and 3.5, where the calculated lipid area remained relatively constant (coefficient of variation: CV < 5%). Beyond this range, the lipid area either decreased (for k > 3.5) due to exclusion of weaker signals, or increased (for k < 2.5) due to inclusion of noise-related pixels, confirming that the selection of k within the plateau range yields stable and reliable quantification. Based on this analysis, k = 3.0 was selected

as the optimal threshold, as it fell well within the stable plateau region and provided the best balance between signal detection and noise exclusion. The same fixed threshold was then applied uniformly to all samples and across all histological comparisons to ensure consistency and objectivity.

Single-wavelength PA imaging was not capable of absolute lipid quantification. Nevertheless, relative comparison of lipid signals was feasible after energy normalization, photon-flux correction and background subtraction under matched experimental conditions [S17, S25]. To further validate the biological relevance of the chosen threshold, the lipid areas derived from the PA images at the optimal threshold were compared with the corresponding lipid areas identified by Oil Red O staining. In Fig. S2(b), linear regression analysis revealed a significant linear association between PA-detected lipid area and histology-measured lipid area, with a slope of 0.94 (95% CI: 0.85 to 1.04), intercept of 1.73% (95% CI: -2.11% to 5.58%), $R^2 = 0.92$, $p < 0.0001$, based on 27 plaque sections (n = 27) obtained from 9 mice fed a HFC diet for 4-12 weeks. These results collectively demonstrated that the lipid quantification was robust against threshold variations and that the selected segmentation criterion provided reliable and biologically meaningful measurements, consistent with established practices in quantitative PA imaging, where thresholding based on noise statistics and subsequent sensitivity analysis were standard procedures for ensuring reliable quantification.

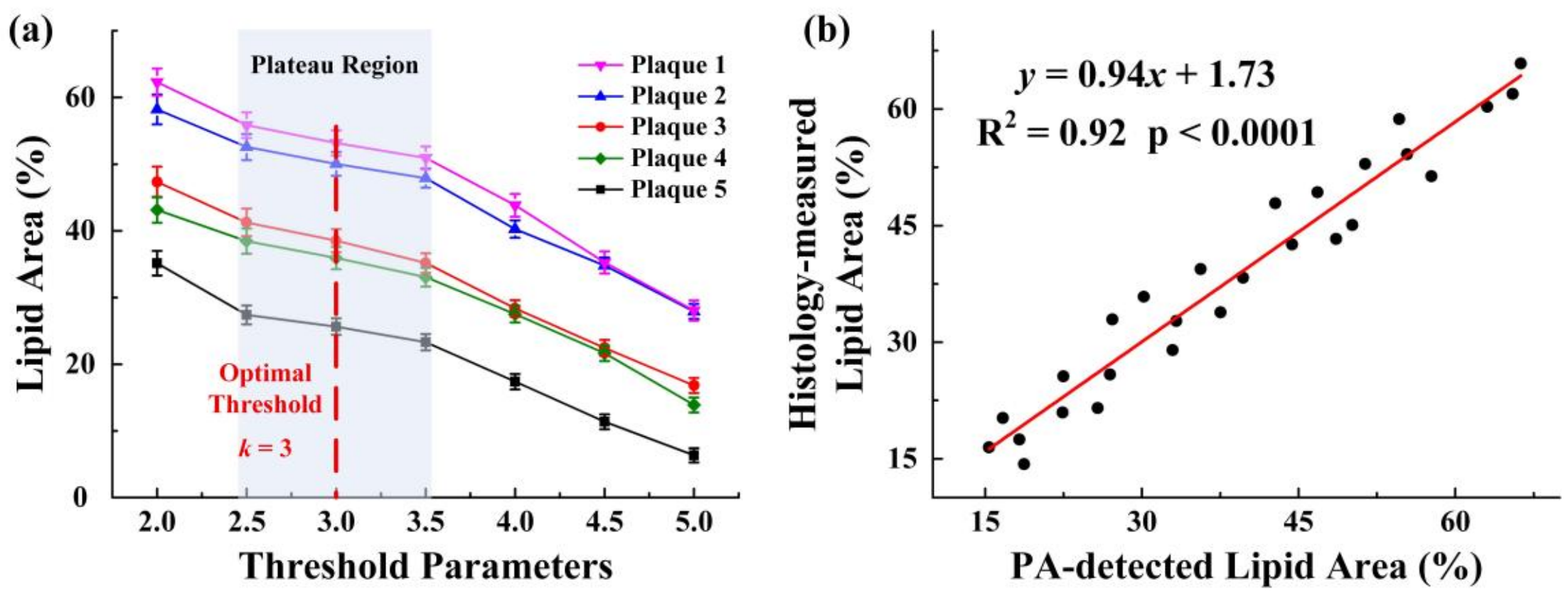

Fig. S2 Threshold sensitivity analysis for lipid area quantification. (a) The plot shows the calculated lipid area (mean ± SD, n = 5) as a function of the segmentation threshold parameter *k*, where the threshold is defined as $T = \mu + k{\cdot}\sigma$. A clear plateau is observed for k values between 2.5 and 5.0, indicating that the lipid area measurement is stable within this range. The final threshold selected for all analyses (k = 3.0) is marked with a red dashed line and falls within this stable plateau region. (b) Linear regression analysis between PA-derived lipid areas and histology-measured lipid areas at the optimal threshold (k = 3.0), demonstrating a strong positive correlation (r = 0.96, 95% CI: 0.94 to 0.99, $p < 0.001$).

4 Phantom experiment

The lipid-gelatin phantoms with precisely predefined lipid concentrations (1%-10%, 10 samples)

were detected for lipid-specific quantitative validation. In Fig. S3, linear regression analysis revealed a significant linear association between lipid concentrations and PA intensity, with a slope of 0.05 a.u./% (95% CI: 0.04 a.u./% to 0.06 a.u./%), intercept of 0.09 a.u. (95% CI: 0.06 a.u. to 0.13 a.u.), $R^2 = 0.97$, $p < 0.0001$, $n = 10$. This result provided direct experimental evidence supporting the quantitative capability of single-wavelength (1700 nm) lipid imaging approach.

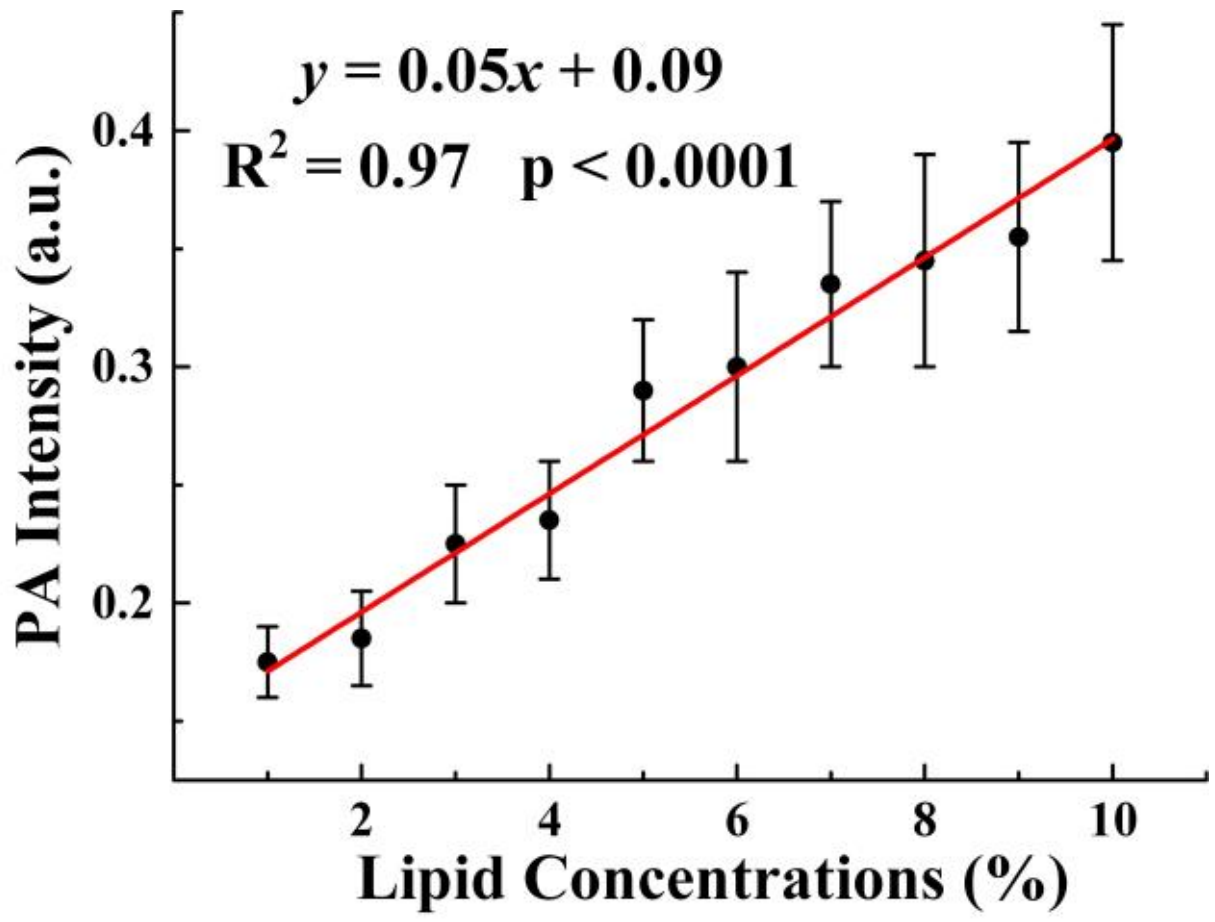

Fig. S3 High positive correlation between lipid concentrations and PA intensity (r = 0.98, 95% CI: 0.942 to 0.998, $p < 0.0001$, n = 10).

S4 Theoretical Derivation of VER

When a modulated laser beam is directed at biological tissue, the light intensity is given by $I = \frac{1}{2} I_0 (1 + \cos \omega t)$, where I_0 is the time-averaged light intensity and ω is the modulation frequency [S34]. Light absorption by the absorber results in a sinusoidal temperature variation in the form of $T = T_0 e^{i\omega t}$ due to the non-radiative transition and then causes thermal expansion and shrinkage as well as PA wave generation based on the thermo-elastic mechanism [S35]. Because of the cyclical changes of light intensity, PA wave, whose dominant frequency is equal to the modulated frequency, is excited periodically. In the above process, the cyclical heating in the local region induces thermal stress, and strain generates due to the stress in the form of force-produced PA waves. Because of the damping effect from viscoelastic biological tissues, it could be found that the strain would be alternated periodically and out of phase with the stress [S5, S36].

In fact, the above-mentioned process is a problem of rheological thermal stress caused by a periodically varied point heat source for an unbounded isotropic viscoelastic solid. To resolve this problem, the radial thermal stress in the spherical coordinate system can be expressed as [S4]:

$$\sigma = -\frac{4G}{r}\frac{\partial \Phi}{\partial r} + \rho \frac{\partial^2 \Phi}{\partial t^2} \tag{1}$$

where G is the Green's function of thermal conduction; Φ is the thermos-elastic displacement potential defined by $\Delta\Phi = \frac{1+\nu}{1-\nu}\alpha T$; α is the coefficient of linear expansion; ν is the Poisson's ratio.

Equation (1) describes the general dynamic radial thermal stress induced by a periodically varying point heat source in an unbounded isotropic viscoelastic medium. To derive the simplified expression **Eq. (2)**, two essential approximations are adopted. First, the quasi-static approximation is applied, where the inertial dynamic term $\rho\frac{\partial^2\Phi}{\partial t^2}$ is neglected, since the inertial effect arising from temperature oscillation is negligible compared with thermoelastic stress within our experimental frequency band. Second, the localized temperature perturbation generated by the point heat source is approximated as a spatially uniform harmonic temperature field $T = T_0 \mathrm{e}^{i\omega t}$.

In addition, it should be emphasized that $\Delta\Phi = \nabla^2\Phi = \frac{1+\nu}{1-\nu}\alpha T$ is a Poisson partial differential equation for Goodier's thermoelastic displacement potential, rather than an algebraic identity $\Phi = \frac{1+\nu}{1-\nu}\alpha T$. Direct substitution of this incorrect algebraic relation into governing equations will lead to erroneous results. For an unconfined infinite elastic solid subjected to uniform temperature variation, the classic quasi-static thermoelastic radial stress solution yields $\sigma(t) = -\frac{E\alpha T}{1-\nu}$, Substituting the harmonic temperature $T = T_0\mathrm{e}^{i\omega t}$, we obtain

$$\sigma(t) = -\frac{E\alpha T_0}{1-\nu}\,\mathrm{e}^{i\omega t} \tag{2}$$

where E is the Young's modulus. The differential constitutive equation of viscoelastic material is

$$P\sigma = Q\varepsilon \tag{3}$$

where ε is the strain and the linear differential operator P and Q are defined as $P = \sum_{k=0}^{m} p_k \frac{d^k}{dt^k}$, $Q = \sum_{k=0}^{m} q_k \frac{d^k}{dt^k}$, respectively. Substitution of **Eq. (2)** into **Eq. (3)** gives the equation:

$$\sum_{k=0}^{m} p_k (i\omega)^k \sigma_0 e^{i\omega t} = \sum_{k=0}^{m} q_k \frac{d^k \varepsilon}{dt^k} \tag{4}$$

Because P and Q are linear differential operators, p_k and q_k are integer, the solution to **Eq. (4)** can be written as $\varepsilon(t) = \varepsilon^* e^{i\omega t}$, where ε^* is the amplitude of the complex strain. Then, **Eq. (4)** becomes:

$$\varepsilon^* = \frac{P^*(i\omega)}{Q^*(i\omega)} \sigma_0 \tag{5}$$

where $P^*(i\omega) = \sum_{k=0}^{m} p_k (i\omega)^k$, and $Q^*(i\omega) = \sum_{k=0}^{m} q_k (i\omega)^k$. The stress-response is

$$\sigma(t) = \frac{Q^*(i\omega)}{P^*(i\omega)} \varepsilon^* e^{i\omega t} = \frac{Q^*(i\omega)}{P^*(i\omega)} \varepsilon(t) \tag{6}$$

The dynamic modulus is the function of the modulation frequency ω:

$$Y^*(i\omega) = Y_1(\omega) + iY_2(\omega) = \frac{Q^*(i\omega)}{P^*(i\omega)} \tag{7}$$

where $Y_1(\omega)$ and $Y_2(\omega)$ are the real-part and imaginary part of $Y^*(i\omega)$ respectively, and they are determined by material constants p_k, q_k and the modulation frequency ω. Thus, by combining **Eqs. (6)** and **(7)**, we have:

$$\varepsilon(t) = [Y^*(i\omega)]^{-1} \sigma(t) = \frac{Y_1(\omega) - iY_2(\omega)}{Y_1^2(\omega) + Y_2^2(\omega)} \sigma(t) \tag{8}$$

The reciprocal of complex compliance can be decomposed via polar representation:

$$[Y^*(i\omega)]^{-1} = \frac{1}{|Y^*(\omega)|} e^{i\delta} = \frac{1}{|Y^*(\omega)|} * \frac{Y_1(\omega) - iY_2(\omega)}{\sqrt{Y_1^2(\omega) + Y_2^2(\omega)}},\ |Y^*(\omega)| = \sqrt{Y_1^2(\omega) + Y_2^2(\omega)} \tag{9}$$

where $|Y^*(\omega)|$ denotes the magnitude of complex compliance and δ represents the phase lag between stress and strain.

The expression in **Eq. (8)** corresponds to the rigorous algebraic form $\frac{Y_1(\omega) - iY_2(\omega)}{Y_1^2(\omega) + Y_2^2(\omega)}$. Equivalently, this can be separated into a magnitude prefactor $\frac{1}{|Y^*(i\omega)|}$ and a normalized phase factor $\frac{Y_1(\omega) - iY_2(\omega)}{\sqrt{Y_1^2(\omega) + Y_2^2(\omega)}}$.

In practice, the amplitude of PA signals is severely disturbed by time-varying optical attenuation, laser

power fluctuation, and variable probe-tissue coupling conditions. These amplitude perturbations hinder reliable quantification of $|Y^*(\omega)|$. In contrast, the phase offset extracted via lock-in amplification is largely immune to multiplicative amplitude artifacts. Therefore, we retain the normalized phase factor to isolate phase dynamics for reconstructing viscoelastic maps, while the magnitude prefactor $\frac{1}{|Y^*(\omega)|}$ is omitted in the simplified expression for imaging reconstruction. Therefore, **Eq. (8)** can be written as

$$\varepsilon(t) = [Y^*(i\omega)]^{-1}\sigma(t) = \frac{Y_1(\omega) - iY_2(\omega)}{\sqrt{Y_1^2(\omega) + Y_2^2(\omega)}}\sigma(t) \tag{10}$$

Equation (1) is the constitutive equation in terms of a stress-strain relationship for the viscoelastic material. The amplitude of the complex strain can be decomposed into two components:

$$\varepsilon^* = \varepsilon' - i\varepsilon'' = \frac{Y_1(\omega) - iY_2(\omega)}{\sqrt{Y_1^2(\omega) + Y_2^2(\omega)}}\sigma_0 \tag{11}$$

Thus,

$$\frac{\varepsilon''}{\varepsilon'} = \frac{Y_2}{Y_1} \tag{12}$$

The strain response in this case becomes:

$$\begin{aligned}\varepsilon(t) = \varepsilon^* \mathrm{e}^{i\omega t} &= (\varepsilon' - i\varepsilon'') * (\cos\omega t + i\sin\omega t) = \varepsilon'\cos\omega t + i\varepsilon'\sin\omega t - i\varepsilon''\cos\omega t - i^2\varepsilon''\sin\omega t \\ &= (\varepsilon'\cos\omega t + \varepsilon''\sin\omega t) + i(\varepsilon'\sin\omega t - \varepsilon''\cos\omega t)\end{aligned} \tag{13}$$

Denoting,

$$\tan\delta = \frac{\varepsilon''}{\varepsilon'} = \frac{Y_2}{Y_1} \tag{14}$$

We obtain the strain response as:

$$
\begin{aligned}
\varepsilon(t) &= (\varepsilon' \cos \omega t + \varepsilon'' \sin \omega t) + i(\varepsilon' \sin \omega t - \varepsilon'' \cos \omega t) \\
&= \varepsilon' \left[\left(\cos \omega t + \frac{\varepsilon''}{\varepsilon'} \sin \omega t \right) + i \left(\sin \omega t - \frac{\varepsilon''}{\varepsilon'} \cos \omega t \right) \right] \\
&= \varepsilon' [(\cos \omega t + \tan \delta \sin \omega t) + i(\sin \omega t - \tan \delta \cos \omega t)] \\
&= \varepsilon' \left[\left(\cos \omega t + \frac{\sin \delta}{\cos \delta} \sin \omega t \right) + i \left(\sin \omega t - \frac{\sin \delta}{\cos \delta} \cos \omega t \right) \right] \\
&= \frac{\varepsilon'}{\cos \delta} [(\cos \delta \cos \omega t + \sin \delta \sin \omega t) + i(\cos \delta \sin \omega t - \sin \delta \cos \omega t)] \\
&= \frac{\varepsilon'}{\cos \delta} [\cos(\omega t - \delta) + i \sin(\omega t - \delta)] = \frac{\varepsilon'}{\cos \delta} \mathrm{e}^{i(\omega t - \delta)} = \varepsilon_A \mathrm{e}^{i(\omega t - \delta)}
\end{aligned}
\tag{15}
$$

For the Kelvin-Voigt model, the complex modulus is defined as $Y^* = E + i\omega\eta$. Complex compliance is the reciprocal of complex modulus:

$$
\varepsilon^* = \frac{1}{Y^*} \sigma_0 = \frac{1}{E + i\omega\eta} \sigma_0 = \frac{E - i\omega\eta}{E^2 + \eta^2 \omega^2} \sigma_0 = \varepsilon' - i\varepsilon'' \tag{16}
$$

By separating real and imaginary terms, the storage strain component under harmonic stress excitation $\sigma(t) = \sigma_0 \, \mathrm{e}^{i\omega t}$ reads

$$
\varepsilon' = \frac{E}{E^2 + \eta^2 \omega^2} \sigma_0 \tag{17}
$$

The thermally induced stress amplitude generated by periodic PA heating follows quasi-static thermoelastic theory:

$$
\sigma_0 = -\frac{E \alpha T_0}{1 - \nu} \tag{18}
$$

Substitute σ_0 into the expression for ε':

$$
\varepsilon' = \frac{E}{E^2 + \eta^2 \omega^2} * \left(-\frac{E \alpha T_0}{1 - \nu}\right) = \frac{-E^2 \alpha T_0}{(1 - \nu)(E^2 + \eta^2 \omega^2)} \tag{19}
$$

Combining with the $\varepsilon_A = \varepsilon' / \cos \delta$, we obtain

$$
\varepsilon_A = \frac{\varepsilon'}{\cos \delta} = -\frac{E \alpha T_0}{(1 - \nu) \cos \delta} \cdot \frac{E}{E^2 + \eta^2 \omega^2} \tag{20}
$$

where ε_A is the amplitude of complex dynamic strain, which is determined by the amplitude of dynamic stress, material constant and modulation frequency. The physical significance of **Eq. (15)** indicates that δ is the phase delay of the strain response to the stress.

Considering the generation procedure of PA signal, the rheological Kelvin-Voigt model is chosen to represent the biological tissue with viscoelasticity [S4, S36]. The rheological Kelvin-Voigt model consists of a spring and a dashpot model. Although it is not an absolute viscoelastic solid, it could describe the creep process under the change of stress. In this model, the dynamic modulus can be expressed as:

$$Y_1 = E \text{ , } Y_2 = \eta\omega \quad \textbf{(21)}$$

Combining the **Eq. (14)** and **Eq. (21)**, we can obtain:

$$\delta = arc\tan\frac{Y_2}{Y_1} = arc\tan\frac{\eta\omega}{E} \quad \textbf{(22)}$$

From **Eq. (22)**, we can obtain the viscosity-elasticity ratio (η/E).

S5 Assessment workflow and classification outcomes of plaques

The plaque classification (lipid plaques, thick-cap plaques and thin-cap fibrous plaques) was not based on the duration of high-fat/high-cholesterol (HFC) diet. Instead, each plaque-bearing cross-section was independently categorized, as detailed below:

(1) Observers: Two independent observers examined each cross-section blinded to diet duration and animal identity.

(2) Prespecified criteria: This study classifies plaques into the following categories:

Lipid plaque: Lipid deposition without a fibrous cap;

Thick-cap fibrous plaque: Fibrous cap thickness ≥ 65 μm;

Thin-cap fibrous plaque: Fibrous cap thickness < 65 μm.

(3) Interobserver agreement: Two independent observers, blinded to the imaging results, classified each plaque according to the histological findings. Interobserver agreement was assessed using Cohen's kappa coefficient (κ). The κ value was 0.94, indicating almost perfect agreement (95% CI: 0.89-1.00). Disagreements were resolved by consensus to obtain the final classification.

Important: Classification was based solely on histological appearance, not on the week of sacrifice. Diet duration was not used to assign plaque type.

For each diet duration, the number of mice and the number of cross-sectional lesions meeting each histopathological category. Figure S4 clearly shows which plaque types were present at each time point, without presupposing that time defines type.

Diet Duration (Weeks)	Mice (n)	Lipid Plaque (n lesions)	Thick-cap Fibrous Plaque (n lesions)	Thin-cap Fibrous Plaque (n lesions)	Total lesions (n)
**0**	3	0	0	0	0
**4**	3	6	0	0	6
**8**	3	9	0	0	9
**12**	3	12	0	0	12
**20**	3	0	14	1	15
**24**	3	0	12	3	15
**Total**	18	27	26	4	57

Fig. S4 Histopathological classification of plaques (all lesions). Each lesion was defined as a plaque region within a single cross-section. All classifications were performed blinded to diet duration and animal identity.

S6 Sample source for statistical analysis

6.1 Lipid plaque linear regression

n = 30 cross-sections selected from mice at 0-12 weeks of HFC diet (i.e., 0, 4, 8, and 12 weeks; 3 mice per time point; multiple non-adjacent cross-sections obtained from each mouse). Notably, the linear analyses for lipid plaques consisted of two parts: the correlation between cross-sectional lipid core area and viscosity-elasticity ratio (VER), and the correlation between macrophage content and VER. Both datasets were derived from approximately identical cross-sections. The detailed sources of cross-sectional data are summarized in Fig. S5.

Diet Duration (Weeks)	Animal ID (Quantity, n)	Aortas (n)	Cross-sections (n)	Image/Plaque Regions (n)	Aortic Segments	Plaque Types	PPI/Rheometer	Histological Staining
0	1 (1)	1	1	1	1 Aortic root	Normal	Y/N	Oil Red O<br>Masson<br>F4/80
	2 (1)	1	1	1	1 Thoracic aorta	Normal	Y/N	Oil Red O<br>Masson<br>F4/80
	3 (1)	1	1	1	1 Abdominal aorta	Normal	Y/N	Oil Red O<br>Masson<br>F4/80
4	4 (1)	1	2	2	2 Aortic root	Lipid plaques	Y/N	Oil Red O<br>Masson<br>F4/80
	5 (1)	1	2	2	1 Aortic root;<br>1 Thoracic aorta	Lipid plaques	Y/N	Oil Red O<br>Masson<br>F4/80
	6 (1)	1	2	2	2 Aortic root	Lipid plaques	Y/N	Oil Red O<br>Masson<br>F4/80
8	7 (1)	1	3	3	1 Aortic root;<br>1 Thoracic aorta;<br>1 Abdominal aorta	Lipid plaques	Y/N	Oil Red O<br>Masson<br>F4/80
	8 (1)	1	3	3	1 Aortic root;<br>2 Thoracic aorta	Lipid plaques	Y/N	Oil Red O<br>Masson<br>F4/80
	9 (1)	1	3	3	2 Aortic root;<br>1 Thoracic aorta	Lipid plaques	Y/N	Oil Red O<br>Masson<br>F4/80
12	10 (1)	1	4	4	1 Aortic root;<br>2 Thoracic aorta;<br>1 Abdominal aorta	Lipid plaques	Y/N	Oil Red O<br>Masson<br>F4/80
	11 (1)	1	4	4	2 Aortic root;<br>1 Thoracic aorta;<br>1 Abdominal aorta	Lipid plaques	Y/N	Oil Red O<br>Masson<br>F4/80
	12 (1)	1	4	4	1 Aortic root;<br>1 Thoracic aorta;<br>2 Abdominal aorta	Lipid plaques	Y/N	Oil Red O<br>Masson<br>F4/80

Fig. S5 Distribution of sample sizes for lipid plaque cross-sections. Y = yes; N = no.

6.2 Fibrous plaque linear regression

n = 30 cross-sections selected from mice at 20 and 24 weeks of HFC diet (3 mice per time point; multiple non-adjacent cross-sections obtained from each mouse). Similarly, linear analyses for fibrous plaques also comprised two correlations: the relationship between fibrous cap thickness and VER, and that between macrophage content and VER. Both datasets were derived from approximately identical cross-sections. The detailed sources of cross-sectional data are summarized in Fig. S6.

Diet Duration (Weeks)	Animal ID (Quantity, n)	Aortas (n)	Cross-sections (n)	Image/Plaque Regions (n)	Aortic Segments	Plaque Types	PPI/Rheometer	Histological Staining
20	13 (1)	1	5	5	2 Aortic root;<br>2 Thoracic aorta;<br>1 Abdominal aorta	Fibrous plaques	Y/N	Oil Red O<br>Masson<br>F4/80
	14 (1)	1	5	5	1 Aortic root;<br>2 Thoracic aorta;<br>2 Abdominal aorta	Fibrous plaques	Y/N	Oil Red O<br>Masson<br>F4/80
	15 (1)	1	5	5	1 Aortic root;<br>2 Thoracic aorta;<br>2 Abdominal aorta	Fibrous plaques	Y/N	Oil Red O<br>Masson<br>F4/80
24	16 (1)	1	5	5	1 Aortic root;<br>2 Thoracic aorta;<br>2 Abdominal aorta	Fibrous plaques	Y/N	Oil Red O<br>Masson<br>F4/80
	17 (1)	1	5	5	2 Aortic root;<br>2 Thoracic aorta;<br>1 Abdominal aorta	Fibrous plaques	Y/N	Oil Red O<br>Masson<br>F4/80
	18 (1)	1	5	5	1 Aortic root;<br>2 Thoracic aorta;<br>2 Abdominal aorta	Fibrous plaques	Y/N	Oil Red O<br>Masson<br>F4/80

Fig. S6 Distribution of sample sizes for fibrous plaque cross-sections. Y = yes; N = no.

6.3 Bland-Altman analysis

n = 20 cross-sections selected from different HFC diet time points. Furthermore, the cross-sections included in this analysis differed from those used in the earlier linear analyses, as histological staining was performed following the measurements on cross-sections for linear analyses. And cross-sections for Bland-Altman analysis were randomly sampled from eligible specimens compatible with both PPI and rheometer, incorporating plaques obtained from different time points and vascular regions. This sampling strategy is critical for two main reasons. First, random sampling within the eligible specimen pool helps minimize selection bias. Second, the inclusion of plaques from multiple time points and vascular regions ensures that the specimens cover diverse pathological stages and morphological features of atherosclerotic lesions. Accordingly, the consistency results derived from Bland-Altman analysis can better represent the overall performance of the PPI technique rather than only reflecting a limited subset of plaque types. The detailed sources of cross-sectional data are summarized in Fig. S7.

Diet Duration (Weeks)	Animal ID (Quantity, n)	Aortas (n)	Cross-sections (n)	Image/Plaque Regions (n)	Aortic Segments	PPI/Rheometer	Histological Staining
0	1 (1)	1	1	1	1 Aortic root	Y/Y	N
	2 (1)	1	1	1	1 Thoracic aorta	Y/Y	N
	3 (1)	1	1	1	1 Abdominal aorta	Y/Y	N
4	4 (1)	1	1	1	1 Aortic root	Y/Y	N
	5 (1)	1	0	0	—	Y/Y	N
	6 (1)	1	1	1	1 Thoracic aorta	Y/Y	N
8	7 (1)	1	2	2	1 Aortic root;<br>1 Thoracic aorta	Y/Y	N
	8 (1)	1	1	1	1 Aortic root	Y/Y	N
	9 (1)	1	0	0	—	Y/Y	N
12	10 (1)	1	1	1	1 Thoracic aorta	Y/Y	N
	11 (1)	1	1	1	1 Abdominal aorta	Y/Y	N
	12 (1)	1	2	2	1 Aortic root;<br>1 Abdominal aorta	Y/Y	N
20	13 (1)	1	2	2	1 Thoracic aorta;<br>1 Abdominal aorta	Y/Y	N
	14 (1)	1	1	1	1 Aortic root	Y/Y	N
	15 (1)	1	1	1	1 Thoracic aorta	Y/Y	N
24	16 (1)	1	1	1	1 Abdominal aorta	Y/Y	N
	17 (1)	1	1	1	1 Thoracic aorta	Y/Y	N
	18 (1)	1	2	2	1 Aortic root;<br>1 Abdominal aorta	Y/Y	N

Fig. S7 Distribution of sample sizes for Bland-Altman analysis. Y = yes; N = no.

Figures S5-S7 enable transparent traceability of each cross-section and eliminates confusion regarding sample size notation.

S7 Confusion matrix results

As requested, we performed a classification analysis (lipid plaques and thick-cap fibrous plaques) using detection parameters of PPI and PAI as predictors. Specifically, we used a logistic regression classifier with VER (PPI) and lipid core relative area (LCRA, PAI) as input features. The optimal

threshold was selected by maximizing the Youden's index [S37]. To reduce overfitting given the limited sample size, model performance was evaluated using leave-one-out cross-validation [S38]. The confusion matrix was computed to enumerate true-positive, false-positive, true-negative, and false-negative samples, thereby delineating the misclassification distribution across plaque types.

The confusion matrix of combined VER and LCRA features for lipid plaque versus thick-cap fibrous plaque discrimination was shown in Table S5. At the optimal threshold determined by maximizing Youden's index (VER = 2.53 × 10^{-5} s; LCRA = 60.38%), the numbers of true-positive, false-negative, false-positive, and true-negative samples were TP (n = 25), FN (n = 2), FP (n = 3), and TN (n = 23), respectively. The classifier yielded a sensitivity of 92.59%, a specificity of 88.46%, a positive predictive value (PPV) of 89.29%, and a negative predictive value (NPV) of 92.00%. Consistent with the partial overlap of VER and LCRA observed between the two plaque types, certain misclassifications were noted in the classification outputs.

Table S5. Confusion matrix: classification of lipid plaques and thick-cap fibrous plaques by VER and LCRA two features.

	**Predicted Lipid Plaque**	**Predicted thick-cap Fibrous Plaque**	**Total**
**Actual Lipid Plaque**	25	2	27
**Actual thick-cap Fibrous Plaque**	3	23	26
**Total**	28	25	53

While the confusion matrix of only VER feature for lipid plaque versus thick-cap fibrous plaque discrimination was shown in Table S6. At the optimal threshold determined by maximizing Youden's index (VER = 2.64 × 10^{-5} s), the numbers of true-positive, false-negative, false-positive, and true-negative samples were TP (n = 22), FN (n = 5), FP (n = 6), and TN (n = 20), respectively. The classifier yielded a sensitivity of 81.48%, a specificity of 76.92%, a PPV of 78.57%, and a NPV of 80.00%. On the other hand, the confusion matrix of only LCRA feature for lipid plaque versus thick-cap fibrous plaque discrimination was shown in Table S7. At the optimal threshold determined by maximizing Youden's index (LCRA = 63.75%), the numbers of true-positive, false-negative, false-positive, and true-negative samples were TP (n = 21), FN (n = 6), FP (n = 8), and TN (n = 18), respectively. The classifier yielded a sensitivity of 77.78%, a specificity of 69.23%, a PPV of 72.41%,

and a NPV of 75.00%.

Table S6. Confusion matrix: classification of lipid plaques and thick-cap fibrous plaques by VER feature.

	Predicted Lipid Plaque	Predicted thick-cap Fibrous Plaque	Total
**Actual Lipid Plaque**	22	5	27
**Actual thick-cap Fibrous Plaque**	6	20	26
**Total**	28	25	53

Table S7. Confusion matrix: classification of lipid plaques and thick-cap fibrous plaques by LCRA feature.

	Predicted Lipid Plaque	Predicted thick-cap Fibrous Plaque	Total
**Actual Lipid Plaque**	21	6	27
**Actual thick-cap Fibrous Plaque**	8	18	26
**Total**	29	24	53

References

[S1] C. Marceau, G. Gingras, S. Thomas, Y. Kassimi, and B. Witzel, "Energy-phase coupling inside sapphire-based f-2f nonlinear interferometers from 800 to 1940 nm", *Appl. Opt.* **53**(5), 898-901 (2014) [doi:10.1364/AO.53.000898].

[S2] Y. Xu, S. Wu, and W. Zheng, "Laser energy deposition and its dynamic uniformity for direct-drive capsules", *Phys. Plasmas* **22**(4), 225 (2015) [doi:10.1063/1.4919032].

[S3] R. Gao, Y. Liu, S. Qi, L. Song, J. Meng, and C. Liu, "Influence mechanism of the temporal duration of laser irradiation on photoacoustic technique: A review", *J. Biomed. Opt.* **29**(S1), S11530 [doi:10.1117/1.JBO.29.S1.S11530].

[S4] K. Maslov and L. V. Wang, "Photoacoustic imaging of biological tissue with intensity-modulated continuous-wave laser", *J. Biomed. Opt.* **13**(2), 024006 (2008) [doi:10.1117/1.2904965].

[S5] G. Gao, S. Yang, and D. Xing, "Viscoelasticity imaging of biological tissues with phase-resolved photoacoustic measurement", *Opt. Lett*. 36(17), 3341-3343 (2011) [doi:10.1364/OL.36.003341].

[S6] S. J. Kleis, and L. A. Sanchez, "Dependence of speed of sound on salinity and temperature in concentrated NaCl solutions", *Sol. Energy* **45**(4), 201-206 (1990) [doi:10.1016/0038-092X(90)90087-S].

[S7] Y. Slobodkin and O. Katz, "Computational wave-based photoacoustic imaging through an unknown thick aberrating layer", *Photoacoustics* **36**, 100584 (2024) [doi:10.1016/j.pacs.2024.100584].

[S8] Q. Q. Mao, Y. J. Feng, C. Tao, and X. J. Liu, "Ultrasound-assisted aberration correction of transcranial photoacoustic imaging based on angular spectrum theory", *Photoacoustics* **40**, 100665 (2024) [doi:10.1016/j.pacs.2024.100665].

[S9] M. S. Osman, H. Y. Chen, K. Creamer, J. Minotto, J. Y. Liu, S. Mirg, J. Christian, X. L. Bai, S. Agrawal, and S. R. Kothapalli, "A novel matching layer design for improving the performance of transparent ultrasound transducers", *IEEE Trans. Ultrason. Ferroelectr. Freq. Control.* **69**(9), 2672-2680 (2022) [doi:10.1109/TUFFC.2022.3195998].

[S10] T. Lee, Q. C. Li, and L. J. Guo, “Out-coupling of longitudinal photoacoustic pulses by mitigating the phase cancellation”, *Sci. Rep.* **6**, 21511 (2016) [doi:10.1038/srep21511].

[S11] A. G. Gertsch, M. Jaeger, N. L. Bush, M. Frenz, and J. C. Bamber, “Influence of inhomogeneity of optical absorbers on optoacoustic signals: A comparison between experiment and theory”, *Proc. SPIE* **7265**, 72650L (2009) [doi:10.1117/12.813701].

[S12] D. Cui, J. Mi, Z. Zhang, X. Su, X. Sun, Y. Shi, and S. Yang, “Ultrafast photoacoustic cavitation pumped by picosecond laser for high-efficient and long-term shockwave theranostics”, *Photoacoustics* **33**, 100546 (2023) [doi:10.1016/j.pacs.2023.100546].

[S13] K. Kagata, S. Torii, R. Uehara, S. Kinoshita, and A. Yoshida, “Investigation of thermal diffusivity measurement technique based on photoacoustic signal response under the influence of three-dimesional heat transfer”, *Appl. Therm. Eng.* **258**, 124519 (2025) [doi:10.1016/j.applthermaleng.2024.124519].

[S14] S. I. Azzam and A. V. Kildishev, “Time-domain dynamics of reverse saturable absorbers with application to plasmon-enhanced optical limiters”, *Nanophotonics* **8**(1), 145-151 (2018) [doi:10.1515/nanoph-2018-0139].

[S15] H. Renner, “Nonlinear distortion of optical pulses by self-produced free carriers in short or highly lossy silicon-based waveguides”, *Opt. Express* **20**(23), 25718-25743 (2012) [doi:10.1364/OE.20.025718].

[S16] R. T. Lee, A. J. Grodzinsky, E. H. Frank, R. D. Kamm, and F. J. Schoen, “Structure-dependent dynamic mechanical behavior of fibrous caps from human atherosclerotic plaques”, *Circulation* **83**(5), 1764-1770 (1991) [doi:10.1161/01.cir.83.5.1764].

[S17] T. Feng, Y. Zhu, K. M. Kozloff, B. Khoury, Y. Xie, X. Wang, M. Cao, J. Yuan, D. Ta, and Q. Cheng, “Bone chemical composition assessment with multi-wavelength photoacoustic analysis”, *Appl. Sci.* **10**(22), 8214 (2020) [doi:10.3390/app10228214].

[S18] H. Wang, X. Zhu, X. Weng, L. Deng, Y. Zheng, Z. Shen, H. You, H. Tang, X. Dong, M. Li, S. Bai, J. Dong, and H. He, “Hand-held laser for miniature photoacoustic microscopy: Triggerable, millimeter scale, cost-effective, and functional”, *Photonics Research* **13**(6),1637-1646 (2025) [doi:10.1364/PRJ.553607].

[S19] B. T. Cox, J. G. Laufer, and P. C. Beard, “Quantitative photoacoustic image reconstruction using fluence dependent chromophores”, *Biomed. Opt. Express* **1**(1), 201-208 (2010) [doi:10.1364/BOE.1.000201].

[S20] A. Asadollahi, H. Latifi, M. Pramanik, H. Qazvini, A. Rezaei, H. Nikbakht, and A. Abedi, “Axial accuracy and signal enhancement in acoustic-resolution photoacoustic microscopy by laser jitter effect correction and pulse energy compensation”, *Biomed. Opt. Express* **12**(4), 1834-1845 (2021) [doi:10.1364/BOE.419564].

[S21] M. N. Cherkashin, C. Brenner, and M. R. Hofmann, “Transducer-matched multipulse excitation for signal-to-noise ratio improvement in diode laser-based photoacoustic systems”, *J. Biomed. Opt.* **24**(4), 046001.1-046001.8 (2019) [doi:10.1117/1.JBO.24.4.046001].

[S22] M. Kuriakose, C. D. Nguyen, M. K. A. Singh, and S. Mallidi, “Optimizing irradiation geometry in LED-based photoacoustic imaging with 3D printed flexible and modular light delivery system”, *Sensors* **20**(13), 3789 (2020) [doi:10.3390/s20133789].

[S23] M. Grzegorczyk, S. J. Pogorzelski, A. Pospiech, and K. B. Szmyt, “Monitoring of marine biofilm formation dynamics at submerged solid surfaces with multitechnique sensors”, *Front. Mar. Sci.* **5**, 363 (2018) [doi:10.3389/fmars.2018.00363].

[S24] A. Q. Bauer, R. E. Imothdurft, T. N. Erpelding, L. V. Wang, and J. P. Culver, “Quantitative photoacoustic imaging: Correcting for heterogeneous light fluence distributions using diffuse optical tomography”, *J. Biomed. Opt.* **16**(9), 096016 (2011) [doi:10.1117/1.3626212].

[S25] J. Y. Hwang, S. Wachsmann-Hogiu, V. K. Ramanujan, A. G. Nowatzyk, Y. Koronyo, L. K. Medina-Kauwe, Z. Gross, H. B. Gray, and D. L. Farkas, “Multimodal wide-field two-photon excitation imaging: Characterization of the technique for *in vivo* applications”, *Biomed. Opt. Express* **2**(2), 356-364 (2011) [doi:10.1364/BOE.2.000356].

[S26] J. L. Sandell, and T. C. Zhu, “A review of *in-vivo* optical properties of human tissues and its impact on PDT”, *J. Biophotonics* **4**(11-12), 773-787 (2011) [doi:10.1002/jbio.201100062].

[S27] H. Yoon, G. P. Luke, and S. Y. Emelianov, “Impact of depth-dependent optical attenuation on wavelength selection for spectroscopic photoacoustic imaging”, *Photoacoustics* **12**, 46-54 (2018) [doi:10.1016/j.pacs.2018.10.001].

[S28] F. M. Brochu, J. Brunker, J. Joseph, M. R. Tomaszewski, S. Morscher, and S. E. Bohndiek, “Towards quantitative evaluation of tissue absorption coefficients using light fluence correction in optoacoustic tomography”, *IEEE Trans. Med. Imaging* **36**(1), 322-331 (2017) [doi:10.1109/TMI.2016.2607199].

[S29] P. Wang, H. W. Wang, M. Sturek, and J. Cheng, “Bond-selective imaging of deep tissue through the optical window between 1600 and 1850 nm”, *J. Biophotonics* **5**(1), 25-32 (2011) [doi:10.1002/jbio.201100102].

[S30] Z. Chen, X. Wang, M. Yang, J. Ming, B. Yun, L. Zhang, X. Wang, P. Yu, J. Xu, H. Zhang, and F. Zhang, “An extended NIR -II superior imaging window from 1500 to 1900nm for high-resolution *in vivo* multiplexed imaging based on lanthanide nanocrystals”, *Angew. Chem. Int. Ed.* **62**(49), e202311883 (2023) [doi:10.1002/anie.202311883].

[S31] R. R. Anderson, W. Farinelli, H. Laubach, D. Manstein, A. N. Yaroslavsky, J. G. Iii, K. Jordan, G. R. Neil, M. Shinn, W. Ghandler, G. P. Williams, S. V. Benson, D. R. Douglas, and H. F. Dylla, “Selective photothermolysis of lipid-rich tissues: a free electron laser study”, *Lasers Surg. Med.* **38**(10), 913-919 (2006) [doi:10.1002/lsm.20393].

[S32] Y. Cao, A. Kole, J. Hui, Y. Zhang, J. Mai, M. Aloosh, M. Sturek, and J. Cheng, “Fast assessment of lipid content in arteries *in vivo* by intravascular photoacoustic tomography”, *Sci. Rep.* **8**(1), 2400 (2018) [doi:10.1038/s41598-018-20881-5].

[S33] C. A. Boiangiu, A. Olteanu, A. V. Stefanescu, D. Rosner, and A. I. Egner, “Local thresholding image binarization using variable-window standard deviation response”, *Ann. DAAAM 2010, Proc. 21st Int. DAAAM Symp.* (2010) [doi:doi:http://dx.doi.org/].

[S34] A. Barbora, O. Bohar, A. A. Sivan, E. Magory, A. Nause, and R. Minnes, “Higher pulse frequency of near-infrared laser irradiation increases penetration depth for novel biomedical applications”, *PLoS One* **16**, e0245350 (2021) [doi:10.1371/journal.pone.0245350].

[S35] X. Lian, J. M. Ruddock, and G. J. Diebold, “Photoacoustic transients generated by laser irradiation of thin films”, *Photoacoustics* **3**, 60-63 (2015) [doi:10.1016/j.pacs.2015.02.003].

[S36] F. Yang, Z. Chen, P. Wang, and Y. Shi, “Phase-domain photoacoustic mechanical imaging for quantitative elastography and viscography”, *IEEE Trans. Biomed. Eng.* **71**(8), 2330-2340 (2024) [doi:10.1109/TBME.2024.3368150].

[S37] R. Fluss, F. David, and R. Benjamin, “Estimation of the Youden Index and its associated cutoff point”, *Biom. J.* **47**(4), 458-472 (2005) [doi: 10.1002/bimj.200410135].

[S38] H. Cheng, D J. Garrick, and R L. Fernando, “Efficient strategies for leave-one-out cross validation for genomic best linear unbiased prediction”, *J. Anim. Sci. Biotechnol.* **8**(1), 38 (2017) [doi:10.1186/s40104-017-0164-6].
